# Metal Electrocatalysts for Hydrogen Production in Water Splitting

**DOI:** 10.1021/acsomega.3c07911

**Published:** 2024-01-29

**Authors:** Amir Kazemi, Faranak Manteghi, Zari Tehrani

**Affiliations:** † Research Laboratory of Inorganic Chemistry and Environment, Department of Chemistry, Iran University of Science and Technology, 16846-13114 Tehran, Iran; ‡ The Future Manufacturing Research Institute, Faculty of Science and Engineering, Swansea University, SA1 8EN Swansea, United Kingdom

## Abstract

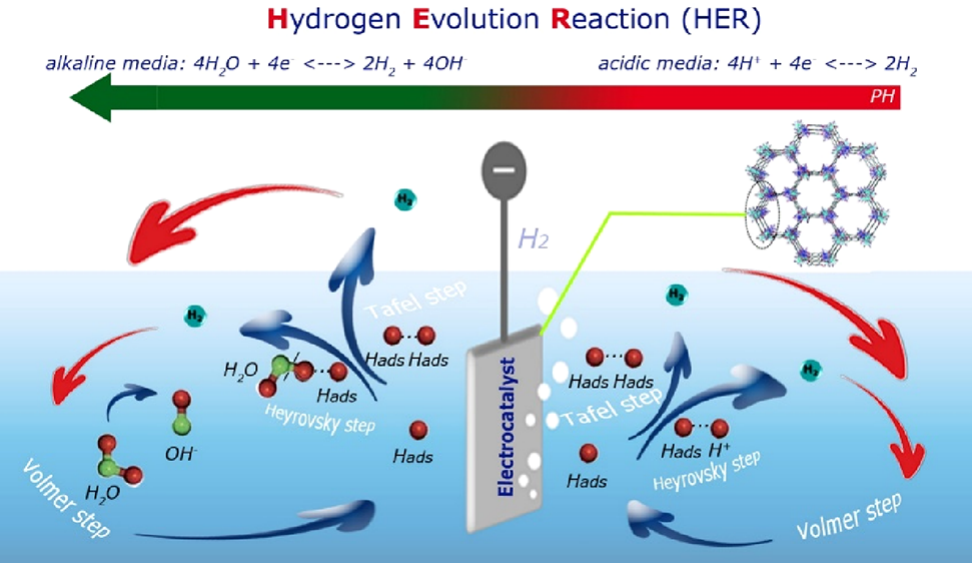

The rising demand for fossil fuels and the resulting pollution have raised environmental concerns about energy production. Undoubtedly, hydrogen is the best candidate for producing clean and sustainable energy now and in the future. Water splitting is a promising and efficient process for hydrogen production, where catalysts play a key role in the hydrogen evolution reaction (HER). HER electrocatalysis can be well performed by Pt with a low overpotential close to zero and a Tafel slope of about 30 mV dec^–1^. However, the main challenge in expanding the hydrogen production process is using efficient and inexpensive catalysts. Due to electrocatalytic activity and electrochemical stability, transition metal compounds are the best options for HER electrocatalysts. This study will focus on analyzing the current situation and recent advances in the design and development of nanostructured electrocatalysts for noble and non-noble metals in HER electrocatalysis. In general, strategies including doping, crystallization control, structural engineering, carbon nanomaterials, and increasing active sites by changing morphology are helpful to improve HER performance. Finally, the challenges and future perspectives in designing functional and stable electrocatalysts for HER in efficient hydrogen production from water-splitting electrolysis will be described.

## 1 Introduction

Today, political and economic crises and global concerns such as declining fossil fuel reserves, environmental pollution, acid rain, global warming, overcrowding, rising consumption, and economic growth are challenging scientists to find appropriate solutions to the world’s energy problems.^1,2^ This problem can only be rectified by replacing green and renewable energy sources.^3^ In recent decades, hydrogen has been called the best and most promising alternative due to its high gravitational energy density, environmental friendliness, and combustion without producing pollutants.^4^ Meanwhile, the energy density of hydrogen is approximately equivalent to 33.5 kWh/kg of usable energy, while this amount of energy for diesel is about 13 kWh/kg.^5^ In other words, the energy of 1 kg of hydrogen, which is used to power the electric motor in fuel cells, is equivalent to a gallon of diesel. In another comparison, diesel has a little lower energy density (45.5 MJ/kg) than gasoline (45.8 MJ/kg). On the other hand, hydrogen has an energy density of approximately 120 MJ/kg, almost three times more than diesel or gasoline.^6^ In the past decade, with the advent of electric propulsion, it has become clear that these propulsions are much more efficient than internal combustion engines, making energy change even more important. In internal combustion engines, almost half of the energy is used to produce heat, while electric vehicle (EV) engines waste less than 10% of energy as heat.^7^ Another appealing feature of hydrogen is price. The diesel price is currently around $3.00 per gallon, and with the recent decline in Iran’s oil production, it is reasonable to expect a further increase in the price of diesel.^8^

Green hydrogen, also known as renewable hydrogen, is hydrogen that is manufactured using only renewable energy, typically by the process of water electrolysis (WE).^9^ The hydrogen evolution reaction (HER) through water splitting has many advantages, including that the reaction can be performed at room temperature and atmospheric pressure. Selective production of oxygen and hydrogen is possible in this process, thus eliminating the gas separation step.^10,11^ Also, the source used (H_2_O) and the products made (O_2_ and H_2_) are environmentally friendly. It is worth noting that despite the tremendous advantages, the HER has limitations due to its high electric power consumption and the use of optimized catalysts.^12^ However, renewable energy application systems, including the HER, depend highly on the appropriate electrocatalyst type.^11,13^ State-of-the-art precious metals and their compounds are active materials for HER and oxygen evolution reactions (Pt for HER, IrO_2_ and RuO_2_ for OER, etc.).^14^ Up to now, Pt is the most efficient catalyst for HER, which requires very small overpotentials even at high reaction rates.^15^ Despite the high efficiency of noble metals, high cost, and scarcity of reserves being barriers to large-scale application, this requires using more affordable and abundant materials for the electrocatalyst.^16,17^

The purpose of this study is to address the pressing environmental concerns regarding energy production through the efficient and sustainable production of hydrogen through water splitting. Hydrogen is a promising source of clean energy, and catalysts play a crucial role in the HER during water splitting. Platinum (Pt) exhibits excellent electrocatalytic activity for HER, but its widespread adoption is hampered by cost considerations. We overcome this challenge by turning to transition metal compounds, which offer both electrocatalytic activity and electrochemical stability. An analysis of the current state and recent advancements in nanostructured electrocatalysts for both noble and non-noble metals will be presented in this investigation. To enhance HER performance by increasing active sites through morphological modifications, we will explore various strategies, such as doping, crystallization control, structural engineering, anion doping in addition to cation doping, and the use of carbon nanomaterials. The final section of the presentation will discuss the challenges that lie ahead and offer future perspectives on how to design functional and stable electrocatalysts for the HER in order to enable efficient hydrogen production by water-splitting electrolysis.

## 2 HER

Currently, hydrogen is mainly used as a raw material in the chemical industry, and its use as a fuel for energy supply has not yet become large-scale. The most economical and common hydrogen generation methods are steam methane reforming (SMR), coal gasification (CG), and WE^18^ as shown in Table 1. The SMR and CG have a price advantage, but they consume fossil fuels and are environmentally damaging. One of the above methods, the WE process, is a green energy method, which produces ultrapure hydrogen (≫99.999) but costs more than conventional methods.^19^ IEA (International Energy Agency) analysis shows that renewable energy’s cost of hydrogen production could fall 30% by 2030.^20^ Although WE can solve environmental problems as a renewable energy production technology, the application of this method is still minimal.^21^ Nevertheless, adequate studies and research are underway to improve this technology to reduce production costs on an industrial scale. In the electrolysis of water, the hydrogen–oxygen bond in a water molecule is broken by electrical power.^22^

**Table 1:** Three Main Criteria for Hydrogen Production on an Industrial Scale

criteria	source	production	reaction
steam methane reforming	methane, steam	H_2_, CO_2_	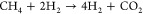
coal gasification	coal, steam	H_2_, CO_2_	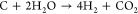
water electrolysis	water	H_2_, O_2_	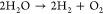

The ability to carry out WE without the utilization of precious metal catalysts in the HER and OER makes it a desirable option in justifying the economics of hydrogen production.^23^ Therefore, developing electrocatalysts with high activity and long-lasting stability to improve the kinetic energy of electrolyte decomposition is critical for potential implementation and useful applications. Electrochemical WE is a promising technology to generate hydrogen fuel from water renewably; this is the process of converting water to pure and stable hydrogen that is made up of two half-cell reactions: the oxygen evolution (OER) and the hydrogen evolution (HER) reaction processes.^24^ Due to the high power consumption in this process, catalysts can reduce the required potential by their performance. Precious metal electrocatalysts (especially Pt-based) show the best performance for the molecular dissociation of water in highly acidic electrolytes, although their HER activities are significantly reduced under alkaline conditions.^25^ As a result, considerable effort has gone into developing effective and sustainable electrocatalysts to replace precious metal catalysts. Chemically, bond-breaking and forming new bonds are effective ways to convert and store energy. According to this principle, WE, also called water splitting, is the process of using electricity to break down water molecules and form their constituent elements.^26^ The process of water splitting without using scarce and expensive electrocatalysts to produce hydrogen has made it an attractive option to make renewable energy more economical in recent years.

### 2.1 Fundamentals of the HER

Water, unlike fossil fuels, is an abundant and renewable resource on Earth, so the hydrogen (H_2_) produced by water splitting can be called the best solution to provide a carrier of green and renewable energy. The HER is a multistep process involving adsorption, reduction, and desorption that takes place on the electrode surface and produces gaseous hydrogen. The three general mechanisms in the HER involve the following reaction steps:
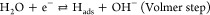
1
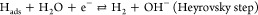
2
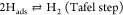
3

The HER mechanism begins with the Volmer step, which is the dissociation of a water molecule and the absorption of H^+^ at the electrode surface (electrocatalysis). The process is then followed by the chemical reaction of the Heyrovsky step or the Tafel step. As per the intrinsic nature of electrocatalysis, the reaction can be carried out with the reaction of two adsorbed H (reaction) or the H adsorption with H^+^ (Heyrovsky reaction) from the electrolyte to release the H_2_ molecule. Whatever the reaction steps, due to the adsorption of H in the reaction, facilitating the adsorption process is the main task of the electrocatalyst.^27^ The free energy from absorption of H in Pt is near to the thermoneutral state (Δ*G*_H*_ ≈ 0). That is why Pt is widely recognized as the greatest HER electrocatalyst available to date. Numerous articles have reported that the Tafel step in the high potential of the HER mechanism is insignificant, and the Volmer–Heyrovsky mechanism is used to carry out the reaction.^13,28^

### 2.2 Acidic and Alkaline Media

**Figure 1:**
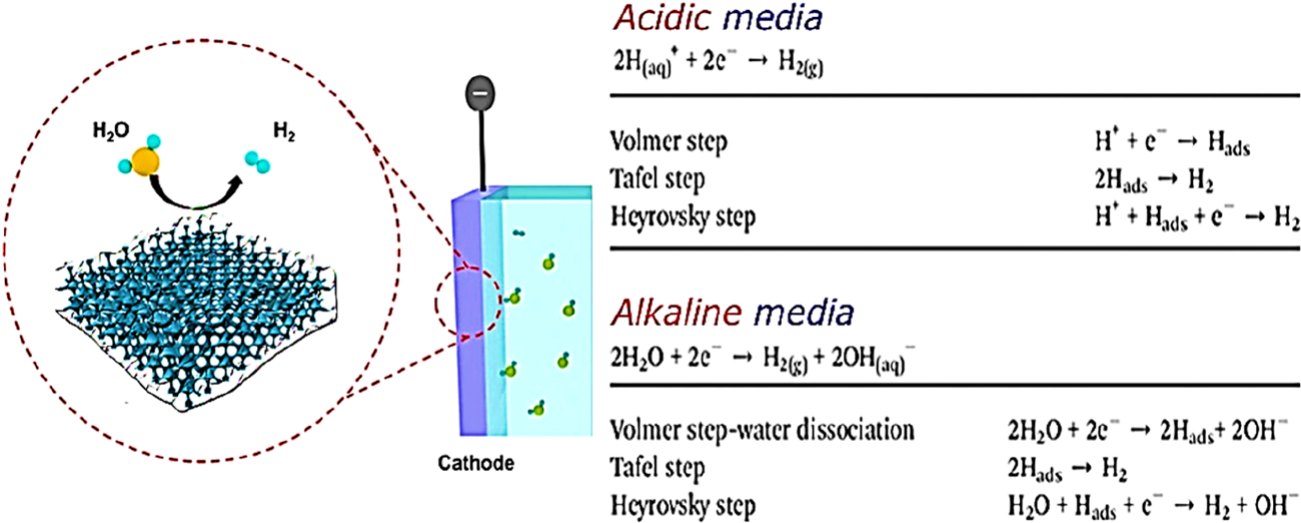
Different mechanisms on the surface of the catalyst in acidic and alkaline environments.

The key half-reaction for hydrogen production in water splitting occurs at the cathode, which involves the transfer of two highly dependent electrons in environmental conditions. The alkaline environment is now the focus of hydrogen development through the HER to substitute clean fuel for different energy systems. Due to an extra water dissociation process, the kinetics of this reaction are sluggish and cause a significant reduction in electrocatalytic performance. Therefore, modern electrocatalysts can perform well in acidic environments.^29^ The electrocatalyst’s effectiveness in an alkaline environment is controlled by theoretical studies based on two factors: water dissociation and then hydrogen-bonding energy. Each electrode with a higher ability to dissociate the water molecule and a better capability to bond to produce a hydrogen atom can therefore be a better electrocatalyst for the HER process in an alkaline environment.^30^ As shown in Figure 1, an electrocatalytic reaction occurring in acidic and alkaline environments follows different mechanisms. In an acidic environment, the mechanism is performed by combining the electrolyte’s proton and one electron from the electrode surface, which is expressed as the Volmer path. The next path is the Tafel path, which is formed by the combination of hydrogen atoms with neighboring atoms. The Heyrovsky path is the latter path, which consists of combining one electron from the electrode surface and another proton from the solution (electrolyte). In alkaline environments, protons are no longer present in electrolytes. Thus, the mechanism begins with dissociating a water molecule, called the Volmer pathway. Then, the Tafel or Hirovsky path continues to produce hydrogen.^31^

Three fundamental phases, one chemical and two electrochemical, make up the electrocatalytic evolution of H_2_ on the electrode surface in an alkaline solution. The first stage is the electrochemical separation of water to generate a hydrogen molecule, which is adsorbed on the surface of the electrode via the Volmer reaction. The next step is an electrochemical process for the hydrogen adsorbed to form H_2_, known as the Heyrovsky reaction, or chemical reaction, known as the Tafel reaction.

It should be noted that the Tafel slope values illustrate the HER process mechanism by describing the potential difference necessary to raise or reduce the current density by 10×. How the current is produced in response to the change of potential applied to the electrode is represented by the Tafel slope. Accordingly, less overpotential is required to obtain high current at a lower Tafel slope (mV/decade). In general, due to the high overpotential of hydrogen, significant electrical energy is required to perform the whole process. Therefore, reducing the cathodic overpotential is one of the challenges in making this process economical. The best way to minimize the cathodic overpotential is to use optimized electrocatalysts such as Pt at the cathode to perform the HER. An acidic medium has less overpotential than an alkaline environment. However, the high cost of membranes and stable electrocatalysts in an acid corrosive environment is one of the disadvantages of this medium.^16,32^

### 2.3 Rate of Reaction

**Figure 2:**
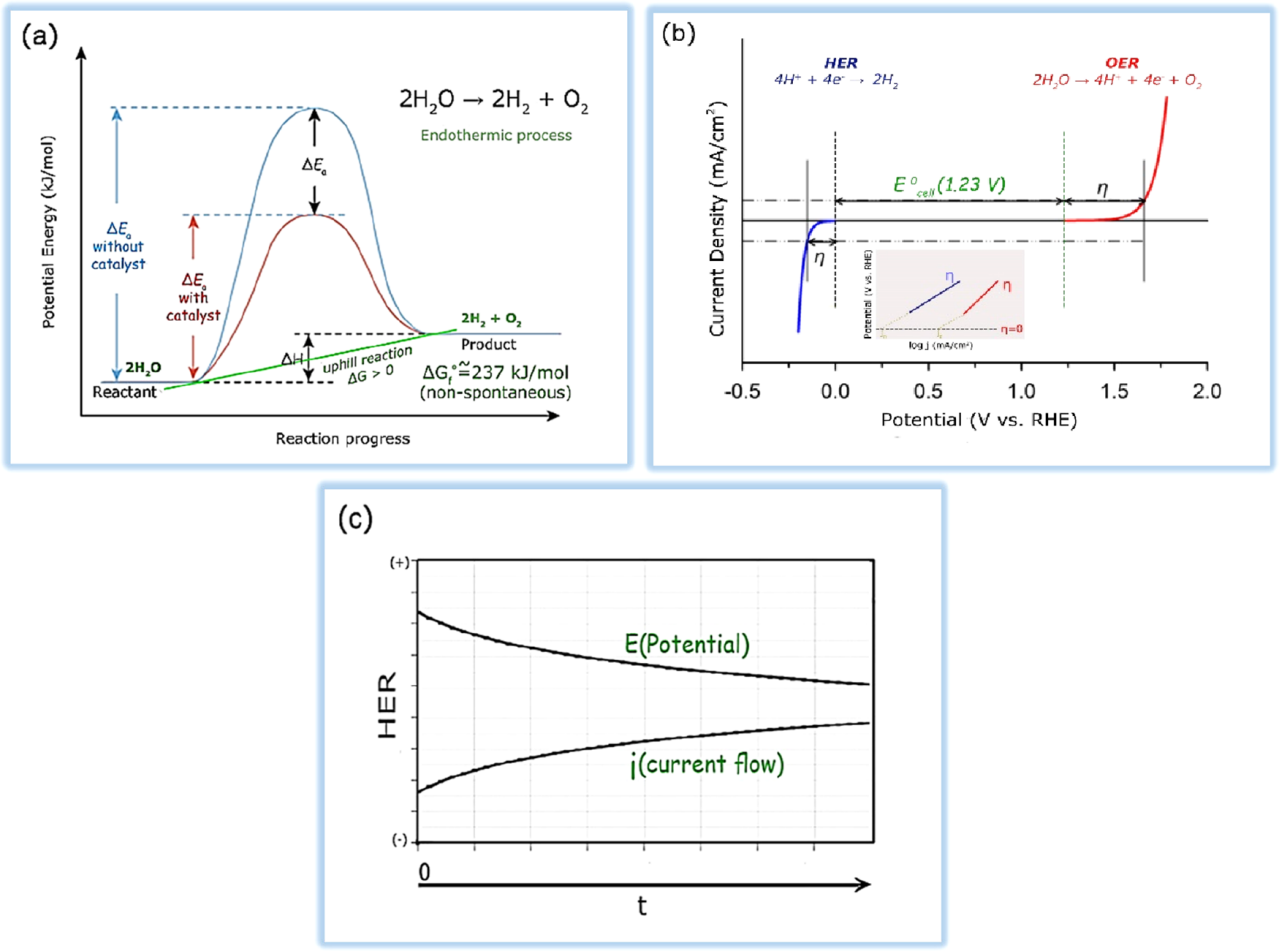
Schematic illustration of the (a) role of the catalyst in reducing the activation energy barrier in the reaction; (b) performance evaluation items of the electrocatalyst, including activity in terms of overpotential, Tafel slope, and exchange current density; (c) performance evaluation items of the electrocatalyst, including stability in terms of current and potential/time curves.

A thermodynamic potential of 1.23 V at 25 °C has been calculated for the electrochemical water-splitting reaction (1 atm). In this reaction, which is an uphill reaction, a large kinetic barrier must be overcome in addition to being expressed by a positive value of *G* (Gibbs free energy). Figure 2a demonstrates that catalysts are essential in reducing the kinetic barrier. This reaction necessitates a greater potential than the thermodynamic potential to overcome the kinetic obstacle (1.23 V). The performance of a catalyst is evaluated based on critical parameters such as activity, stability, and efficiency. The Tafel slope and overpotential can show the activity of the catalysts and the density of the exchange current derived from polarization curves (Figure 2b). Overpotential is a fundamental description for evaluating the activity of electrocatalysts. Overpotential changes and current flow over time are indicators of stability (Figure 2c). The efficiency of an electrocatalyst can be evaluated by comparing experimental results against theoretical predictions with faradaic efficiency and turnover frequency.^33^

The total reaction rate is determined by the free energy of hydrogen adsorption (Δ*G*_H_). If the hydrogen molecule attaches poorly to the surface of the catalyst, the Volmer step (adsorption) will slow down the total reaction rate. However, if the bond between the catalyst surface and the hydrogen is stronger than usual, the desorption is slow, meaning that the Heyrovsky/Tafel phase limits the velocity. Therefore, Δ*G*_H*_ ≈ 0 is a required (but inadequate) prerequisite for an active HER catalyst. With these explanations, it can be concluded that one of the characteristics of an effective catalyst is that the middle bond of the reaction is neither too strong nor too weak.^34,35^

### 2.4 Pseudocapacitance

In the HER process, hydrogen must be adsorbed on the surface of the electrocatalyst in the first step. In the next step, the absorbed hydrogen must be separated from the electrocatalyst surface and returned irreversibly to the electrolyte. This electrocatalyst pseudocapacitive characteristic is critical to HER performance. In hydrogen adsorption, practically all HER electrocatalysts are excellent pseudocapacitors. In order to desorb all of the hydrogen absorbed, a good pseudocapacitor must be highly efficient. Therefore, studying pseudocapacitive performance before the HER potential offers vital information on electrocatalyst efficiency. The optimal pseudocapacitive performance, which is typified by a rectangular shape in cyclic voltammetry (CV), is significantly more closely related to HER electrocatalytic activity.^36^

### 2.5 Volcano Plots

**Figure 3:**
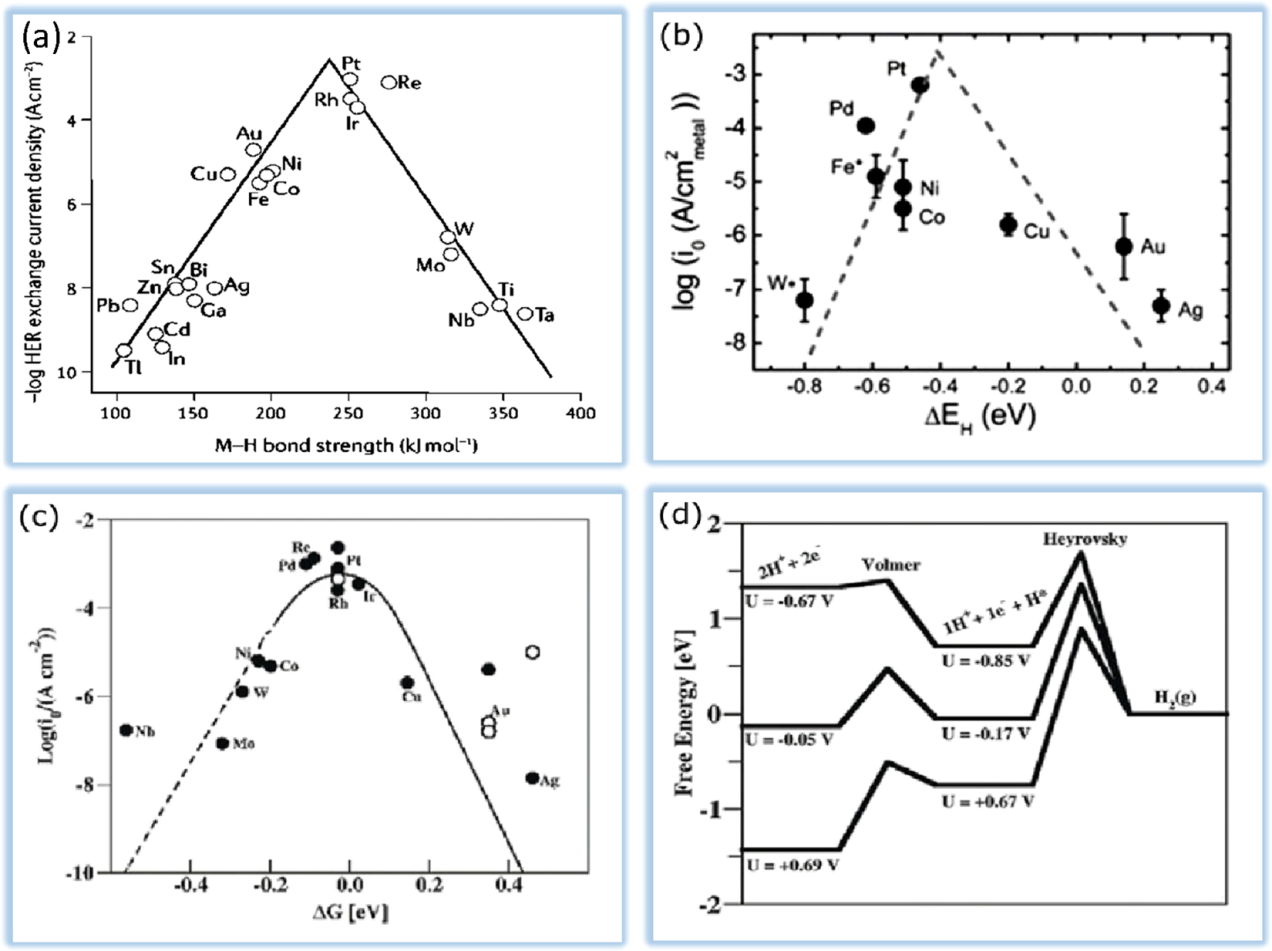
(a) Volcano-type dependence between the exchange current density and metal–hydride (M–H) bond strength. Data taken from ref (41). (b) Exchange current densities, log(*i*_0_), on monometallic surfaces plotted as a function of the calculated HBE. The *i*_0_s for non-Pt metals were obtained by extrapolation of the Tafel plots between −1 and −5 mA cm^–2^ disk to the reversible potential of the HER and then normalization by the ESAs of these metal surfaces. Data taken from ref (38). (c) A volcano plot containing measured *j*_0_ plotted versus the computed Δ*G*_H_ at equilibrium potential in acidic conditions. Data taken from ref (39). (d) Standard free energy diagram for the Volmer–Heyrovsky route HER under different potentials. Data taken from ref (39).

As stated in the previous sections, theoretical simulations prove that HER activity is closely connected to hydrogen adsorption (H_ads_) and that the free energy of hydrogen adsorption (Δ*G*_H_) can accurately characterize hydrogen evolution. Also, the moderate amount of hydrogen-bonding energy in the HER process is of great advantage. The volcano curve, as illustrated in Figures 3a and 3b, can compare the behaviors of various metals in acidic and alkaline environments.^37,38^ Pt is the superior electrocatalyst for HER in both mediums, as demonstrated in the plots, because it has the appropriate hydrogen adsorption energy and hence provides the largest exchange current density. An acidic medium has less overpotential than an alkaline environment. Nevertheless, it is less used due to the corrosion of electrocatalysts. Also, the alkaline environment is critical due to non-noble electrocatalysts. Alkaline water splitting is an excellent way to produce pure hydrogen with high purity. This technology is environmentally friendly and does not emit any carbon dioxide. Pt and Pt-based alloys perform very well in the HER due to their low activation overpotential. As mentioned, HER activity is very often lower in alkaline media than in acidic conditions. This is primarily due to the slowing down of the water dissociation phase. However, alkaline electrolysis is preferred on an industrial scale. It is critical to consider the bonding of hydrogen species and the water dissociation potential when designing electrocatalysts with high alkaline HER performance. According to Figure 3c and Figure 3d,^39^ both the thermodynamic influence of Δ*G*_H_ and the hydrolysis kinetics affect the progress of the reaction. The volcanic plot predicts that metals such as PGMs are on top of the volcano plot with the ideal H binding energy and show the highest activity. Similar to the acidic media, a volcano plot can describe the relationship between H bonding energy values and HER exchange current density at alkaline pH. This description can be defended by experimental studies and density-functional theory (DFT).^40^

### 2.6 Kinetic Isotope Effect (KIE)

An important method for studying chemical reactions is the kinetic isotope effect (KIE). This theory is based on the observation that the rate of a reaction can vary with atom mass. A particle’s mass affects the energy of its vibrational and rotational states, which, in turn, affects the probability of tunneling. Tunneling is a quantum mechanical process that allows particles to pass through potential barriers without having enough energy to overcome them.

Primary and secondary KIEs are the two main types. There is a primary KIE when the difference in mass only exists between the atoms involved in the rate-determining step between two isotopically substituted reactants. A secondary KIE is the ratio of the rates of reaction between two isotopically substituted reactants, where the mass difference is not between the atoms. A KIE can be used to identify the rate-determining step in a reaction. If a primary KIE is observed, then the rate-determining step must involve breaking or forming a bond between the isotopically substituted atoms. Conversely, if a secondary KIE is observed, the rate-determining step must not involve breaking or forming a bond between the isotopically substituted atoms. By providing information about the transition state’s energy, KIEs can also be used to study the mechanism of a reaction. In the case of a primary KIE, the transition state energy for the lighter isotope must be lower than for the heavier isotope. In contrast, if a secondary KIE is observed, then both isotopes must have the same energy of the transition state. Aside from primary and secondary KIEs, equilibrium isotope effects and kinetic secondary isotope effects are also types of KIEs. In equilibrium isotope effects, the equilibrium constants of two isotopically substituted reactions are compared. Kinetic secondary isotope effects are calculated using the ratio of the rate constants of two isotopically substituted reactions in which the difference in mass is not between atoms involved in the rate-determining step but between atoms involved in the equilibrium constant. Chemical reactions can be studied using a KIE. It is possible to use them to identify the rate-determining step, to determine the energy of the transition state, and to study the mechanism of reactions that are difficult to study using other methods. An electron is transferred from the electrode to a hydrogen ion in the electrolyte as part of the HER process. As an electrolyte, both H_2_O and D_2_O have been used to study the KIE of the HER. When D_2_O is used instead of H_2_O in these studies, the rate of the reaction is approximately 6 times faster. Since the D^+^ ion is heavier than the H^+^ ion, it moves through the electrolyte more slowly. Based on the KIE for the HER, the following mechanism can be proposed:

(1) An electron is transferred from the electrode to a hydrogen ion in the electrolyte, forming a hydronium ion.
(2) The hydronium ion dissociates into a proton and water molecule.
(3) The proton tunnels through the water molecule and attacks the surface of the electrode, forming a hydrogen atom.
(4) The hydrogen atom desorbs from the surface of the electrode, forming hydrogen gas.

A KIE indicates that an electron is transferred from the electrode to a hydrogen ion in the electrolyte to determine the rate of the reaction. The isotope effect is much larger for this step than for any of the other steps.^42^

## 3 HER Electrocatalysts Requirements

The disparity between the electrical potentials of each electrode determines the potential of an electrochemical cell. It is impossible to determine the electrical potential of a single electrode; for this purpose, we can set an electrode to zero and use it as a reference. This selected electrode is called the standard hydrogen electrode (SHE). This electrode is a reference for measuring the activity potential of different materials relative to each other. The reduction half-reaction selected for reference is
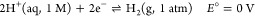
4

In the above relationship, *E*° stands for the standard reduction potential (at a standard temperature of 25 °C and pressure, 1 atm, 1 M for solution), and at all temperatures the voltage is defined as zero (0 V vs RHE). According to theoretical calculations, the potential of the cell along the entire water-splitting path is 1.23 V.^43^ This reduces efficiency and kinetics. Designing and utilizing highly efficient catalysts to reduce the overpotential for OER and HER to produce H_2_ and O_2_ is one efficient way to do this. It is crucial to comprehend the criteria used to assess and contrast the electrocatalytic activity of particular materials and to establish test procedures. Unfortunately, no coordinated effort has been made to develop an integrated test protocol or a way to present quantitative electrocatalytic data. In studies, the performance of electrocatalysts is usually evaluated with the following parameters.^44^

### 3.1 Overpotential, Tafel Slope, and Exchange Current Density

The overpotential (η) component is a fundamental descriptor for assessing the electrocatalysts’ behavior, which is the difference between the theoretical half-reaction reduction potential and the actual cell potential. Suitable electrocatalysts for HER should have a small overpotential because the amount of low overpotential is directly related to the high electrocatalytic activity. Water splitting occurs at a cell potential of 1.23 V (0 V for HER and 1.23 V for the OER). Both HER and OER processes need additional potential, mainly from the intrinsic activation obstacles present. The applied potential must be increased for the reaction to occur.^45,46^ Usually, the overpotential value corresponds to the current density such as 10 mA cm^–2^ (η = 10) and/or 100 mA cm^–2^ (η = 100).

Another approach for measuring HER electrocatalyst activity is to calculate Tafel parameters, the values of which are typically determined by examining the polarization curve as a logarithmic current density diagram (log(*j*)) vs *iR*-compensated overpotential (η). The Tafel slope represents the kinetic relationship between the overpotential and the current density in the electrocatalyst, which is expressed by eq 5:
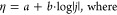
5
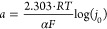
6
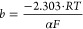
7In this equation, η is the overpotential, and *j* is the current density.

The Tafel slope (*b*) and the exchange current density *j*_0_ are determined by obtaining the current at a potential of zero. As stated, the overpotential is the difference among the electrodes’ potential under the equilibrium potential (*E*_*r*_) and current load (*E_j_*). According to eq 5, the Tafel slope (*b*) is an intensive quantity independent of the surface area of the catalyst, and its value is defined by the rate-determining stage and its location in the reaction process. In contrast, *j*_0_ is a large quantity and depends on the surface area of the catalyst and indicates easy electron transfer with small activation energy. An active electrocatalyst should have a low Tafel slope and a large *j*_0_. A lower Tafel slope suggests a large increase in current density as a function of overpotential variation (i.e., the kinetics of the electrocatalytic reaction are faster).^47,48^

The exchange current density ( *j*_0_) can be defined as the readiness of the electrode to continue the electrochemical reaction, so the higher the exchange current density, the more active the electrode surface. It describes the intrinsic charge transfer exchange current density under an equilibrium state. Increased exchange current densities indicate higher charge transfer rates and better reaction progress.^49^ Therefore, it can be concluded that for a better electrocatalyst, a higher exchange current density and a lower Tafel slope are expected. The scan rate used to obtain the potentiodynamic polarization curve should not be overlooked because this parameter strongly affects the values obtained for the current density and Tafel slope. Potentiostatic method curves or recorded polarization curves should thus be assessed at the slowest scan rate available. The exchange current density ( *j*_0_) indicates the intrinsic interactions and charge transfer activity between the electrocatalyst and the reactant. High exchange current density usually reflects the excellent performance of the HER reaction electrocatalyst. However, it should be noted that obtaining direct exchange current density is challenging, and we can only get the total current density from the Tafel equation experiments.^50^

### 3.2 Electrochemically Active Surface Area (ECSA)

The electrode material’s electrochemical surface area (ECSA) is one of the fundamental phenomena in selecting electrocatalysts, indicating the area of the electrode material available to the electrolyte. It has been proven that it is challenging to measure the electrochemically active surface of any material as an electrode. This surface is used for charge transfer and/or storage in electrochemical cells (galvanic/electrolytic). The electrode surface may be expanded using a variety of techniques. These include using nanostructures, cyclic voltammetry (CV), and linear sweep voltammetry (LSV).^51^

### 3.3 Faradaic Efficiency

Faraday efficiency (also known as current efficiency) is another parameter used to assess the activity of the HER electrocatalyst. In place of the side reaction, faradaic efficiency (FE) calculates the amount of charges (electrons) in the desired reaction. In the HER process, FE is the ratio of experimentally identified H_2_ compared to the theoretical quantity H_2_. This ratio of experimental H_2_ to theoretical H_2_ is calculated using a current density based on 100% faradaic output. The faradaic efficiency is calculated under two parameters: the total amount of hydrogen produced *n*H_2_ (mol) and the total amount of charge *Q* (C) transferred from the cell using eq 7. The total charge is determined by integrating the current, and the total amount of hydrogen generated is determined by using gas chromatography (GC) or the water displacement method.^52^
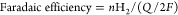
8*F* is the Faraday constant, ≅96 500 C/mol.

### 3.4 Turnover Frequency

The turnover frequency (TOF) of a catalytic center quantifies its particular activity by the number of reactants transformed to the chosen product per unit time. The amount of TOF for HER and OER is calculated based on the following equations:
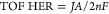
9
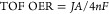
10where *A* represents the area of the working electrode and *J* is the current density; the number 2 represents the electrons for H_2_/mol, and the number 4 represents the electrons for O_2_/mol; also, *n* embodies the mole number of active sites, and *F* is the Faraday constant.

In experiments, measuring TOF is not easy for most solid-state catalysts. In this case, the catalyst’s surface atoms are not catalytically active or uniformly available. A presence of HER bubbles on the electrode surface causes an excessive rise in potential and disturbs the active surface. Admittedly, TOF values cannot provide much accuracy, but they can still assist in linking catalyst activity.^53^

### 3.5 Hydrogen-Bonding Energy

In the hydrogen evolution reaction (HER), an electrochemical process that generates hydrogen gas from water splitting, hydrogen-bonding energy (HBE) plays a crucial role. Hydrogen bond energy describes the strength of hydrogen atoms’ interaction with neighboring atoms, usually oxygen atoms in water or on the catalyst surface. The interaction affects the overall kinetics of the HER, affecting the rate of hydrogen evolution and the stability of adsorbed hydrogen species. Adsorption of hydrogen intermediates on the catalyst surface is made easier by a strong hydrogen bond between hydrogen atoms and the catalyst surface. If the HBE is too strong, it can prevent hydrogen molecules from desorbing, reducing hydrogen evolution efficiency. In order to achieve efficient and sustainable HER, an optimal HBE must be in place. Numerous techniques have been used to study the effects of HBE on HER activity, including theoretical calculations, spectroscopic studies, and electrochemical measurements. Catalysts with a moderate HBE typically exhibit higher HER activity than those with a too weak or too strong HBE, according to these studies. A moderate HBE allows for efficient electron transfer and hydrogen desorption without hindering hydrogen intermediate formation. HER activity is also affected by the distribution of HBE sites on the catalyst surface in addition to the strength of the HBE. The uniform distribution of HBE sites promotes the formation of stable intermediates and efficient hydrogen evolution by providing hydrogen atoms with suitable binding sites. In order to develop efficient HER catalysts, it is essential to optimize HBE. Researchers can design catalysts with the desired HBE properties to achieve high HER rates and stability by understanding the relationship between HBE and HER activity. The development of sustainable hydrogen production technologies depends on this. In order to achieve efficient HER, an optimal HBE is necessary. Catalysts with a moderate HBE typically exhibit higher HER activity than those with either a weak or a strong HBE. A moderate HBE promotes the overall HER process by stabilizing adsorbed hydrogen species and facilitating their desorption.

#### Relationship between HBE and HER Activity

A complex relationship exists between the strength of the HBE and the activity of the HER. Strong HBEs can indeed stabilize adsorbed hydrogen species, but they can also hinder hydrogen desorption if they are too strong. Due to the strong HBE, hydrogen molecules cannot break away from the catalyst surface. Thus, the catalyst becomes saturated with hydrogen atoms, and the overall HER rate decreases.

#### Factors Affecting HBE

There are several factors that can affect the HBE between hydrogen atoms and the catalyst surface, such as the following:

(1) Nature of the catalyst surface: HBE can be significantly affected by the type of metal or material used for the catalyst surface. Catalysts containing a high density of oxygen-containing groups, such as hydroxides and oxides, tend to exhibit stronger HBE than catalysts containing a lower density.
(2) Preparation methods: A catalyst’s preparation method can also affect its HBE. The formation of HBE is more likely to occur in catalysts prepared using methods that introduce defects or roughness into their surfaces.
(3) Electrolyte composition: HBE can also be affected by the electrolyte composition. Electrolytes with higher pH values, for example, tend to form stronger HBE than electrolytes with lower pH values.

#### Optimizing HBE and Impact on Sustainable Hydrogen Production

Developing efficient HER catalysts requires optimizing HBE. Scientists can design catalysts with high HER rates and stability by understanding the factors that influence HBE and tailoring the catalyst surface and preparation methods accordingly. Hydrogen production technologies require efficient HER catalysts for advancement. Optimizing HBE can enhance the performance of HER catalysts, making them more suitable for practical hydrogen production applications, such as fuel cells and hydrogen storage. The hydrogen bonding energy (HBE) determines the stability of adsorbed hydrogen species, the desorption of hydrogen molecules, and the overall efficiency of hydrogen evolution reactions (HER). To develop efficient HER catalysts and realize the potential of sustainable hydrogen production technologies, it is crucial to understand and optimize HBE.

### 3.6 Stability

Another important parameter for choosing the suitable HER electrocatalyst is stability. There are two approaches to assess the stability factor. One is LSV or CV; the other is potentiostatic or galvanostatic electrolysis by long-term chronopotentiometric (CP) or chronoamperometric (CA) tests. This voltammetric method is utilized to compare the overpotential modifications, before and after a certain period of cycles for 1 000–10 000 cyclic voltammograms at a scan rate such as 5–50 mV s^–1^.

In a CV cycle, the higher the number of cycles, the higher the accuracy, and the better it can be to reach a few thousand cycles by a high potential limit of up to 0 V vs RHE. Changes in the parameters of the polarization curve as well as overpotential (at a specific current density) are evaluated before and after the CV cycle to measure stability.^54,55^ The lesser the rate of change, the greater the material’s stability. It is widely acknowledged that assessing a catalyst’s long-term stability is sufficient for around 12 h. However, it is strongly advised that the measures be evaluated for at least 240 h in order for the stability results to be at least somewhat industrially defensible. An HER electrocatalyst must be usable for several thousand hours to be ready for use on a large scale. Accelerated experiments usually last only a few hours at best. Such expedited testing may not give exact operational information for industrial-scale assessment. However, this time is clearly restricted from an industrial viewpoint, and further testing is required before larger-scale implementation.^56,57^

### 3.7 Electrochemical Methods (Three-Electrode Cell)

**Figure 4:**
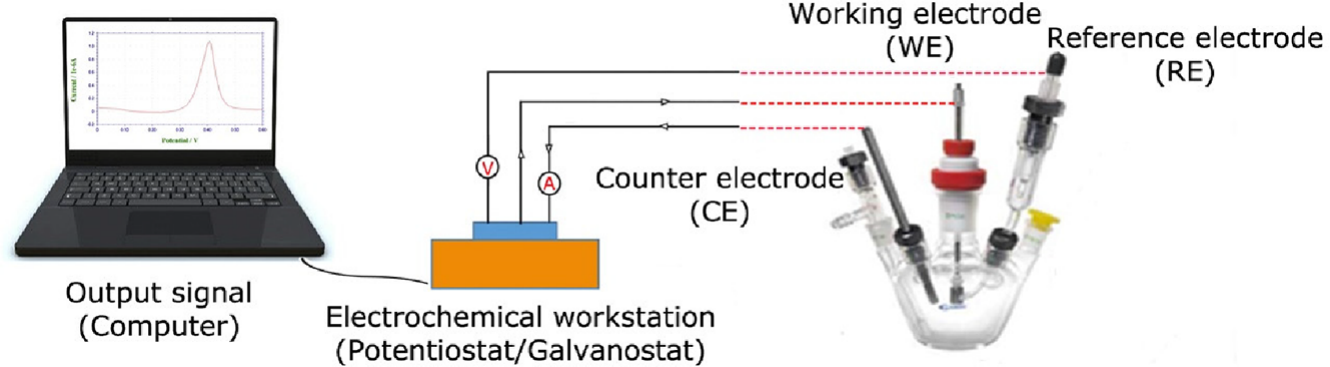
Three-electrode system with working electrodes, counters, and reference electrodes.

In the HER process, a rotating disk work electrode (RDE) is suggested to obtain accurate experimental data. With high accuracy, this electrode can quantify the mass transfer rate and reaction kinetics well. The RDE is a working electrode used in three-electrode systems for voltammetry of electrochemical studies when examining the reaction mechanisms of redox chemistry, among other chemical phenomena.^58^ The electrode is connected to an electric motor with excellent control over the rotation speed of the electrode. The electrode rotates during the test and induces a flux of analyte to the electrode depending on the applied voltage. The structure of this electrode consists of a conductive disk made of a noble metal or glassy carbon surrounded by a nonconductive material such as a polymer or an inert resin. According to Figure 4, in an electrochemical system, the working electrode (WE) is often used in conjunction with a counter electrode (CE) and a reference electrode (RE) in a three-electrode system.^54,59^

### 3.8 Measurement Pitfalls and Data Interpretation in HER Studies

Electrochemical measurements play a crucial role in studying the HER and evaluating the performance of catalysts. However, there are several challenges and pitfalls that researchers may encounter during these measurements, which can affect the accuracy and reliability of the obtained data. In this section, we summarize some common pitfalls and provide guidance for addressing them.

#### Electrode Fouling and Surface Contamination

One of the major challenges in HER measurements is electrode fouling and surface contamination. Over time, the electrode surface can become covered with reaction intermediates or byproducts, leading to altered electrochemical behavior and inaccurate measurements. To mitigate this issue, careful electrode preparation, regular cleaning, and appropriate experimental conditions should be employed. Techniques such as cyclic voltammetry and electrochemical surface cleaning can help remove adsorbed species and restore the electrode surface.

#### Electrolyte Effects

The choice of electrolyte can significantly influence HER measurements. Different electrolytes can have varying pH, ionic strength, and composition, resulting in different reaction kinetics and performance of catalysts. It is crucial to carefully select an electrolyte that is suitable for the specific HER system under investigation. Additionally, understanding the effects of electrolyte composition on the reaction mechanism and kinetics is essential for accurate data interpretation.

#### Proper Data Analysis Techniques

Interpreting electrochemical data accurately is crucial for understanding the performance of HER catalysts. Common pitfalls in data analysis include incorrect baseline subtraction, improper fitting models, and overlooking potential artifacts. Researchers should carefully select appropriate analysis techniques, such as Tafel analysis or electrochemical impedance spectroscopy, and ensure proper data processing and fitting procedures. It is also important to consider potential artifacts, such as capacitive currents or double-layer charging effects, and account for them in the data analysis.

By being aware of these pitfalls and employing appropriate measures, researchers can improve the accuracy and reliability of their electrochemical measurements and data interpretation. Addressing these challenges is essential for advancing our understanding of HER catalysts and developing efficient hydrogen production technologies. Overall, by summarizing the common pitfalls and providing practical guidance for measurement and data interpretation, researchers can navigate through the challenges inherent in HER studies and obtain more accurate and reliable results.

## 4 HER Electrocatalysts

Given the materials investigated as candidate electrocatalysts for HER in the water-splitting process, a coherent method for presentation and comparison is critical.^56,58^ The approach employed in this study is to categorize materials into three major groups:

(1) Noble metals with compounds and alloys
(2) Low-cost transition metal-based materials without precious metals
(3) MOF-derived material-based electrocatalysts

Today, efforts are focused on developing low-cost metal-based electrocatalysts with high stability and performance and are considered an essential and promising candidate for the industrial scale.

### 4.1 Noble Metal-Based Electrocatalysts

The electrocatalytic performance of noble metals, such as Pt group metals (PGMs, including Pt, Pd, Rh, Ru, and Ir), is appealing for HER.^60^ The Volcano diagram shows that these materials are at the top of the curve. However, due to their scarcity and high price, the commercial application of these noble metal electrocatalysts is constrained.^61^ Rational design of catalysts with minimal noble metal load and high usage of other metals is crucial to overcoming this difficulty.^62^ The trend of Pt activity is (111) < (100) < (110) according to the single-crystal aspects with a low Pt index in the HER process. The justification for this trend can be attributed to the presence of H_opd_ (overpotential deposition, weakly adsorbed state) reaction, which is most prevalent at level (110). Therefore, the activity of the Pt surface (110) in the role of electrocatalyst can be greater than that of the other two surfaces.^63^ Therefore, modification and optimization of geometric parameters of noble metal-based electrocatalysts while maintaining activity have been investigated.^64^ The activity of Pt electrocatalysts in an alkaline environment is typically lower than in an acidic environment as a benchmark. This is because the dissociation of water at the Pt surface is inefficient, leading to this electrocatalyst’s weak activity in the electrolyte. Pt coupling with water dissociation promoters is a popular technique for improving the electrocatalytic capabilities of HER in an alkaline environment to compensate for this.^65^ The optimization of geometric parameters and efficient modifications in structure while preserving electrocatalytic activity have been thoroughly explored as a result of the right deployment of Pt-based electrocatalysts. Loading Pt nanoparticles on high-surface carbon is a simple and cost-effective technique to generate active HER electrocatalysts for this purpose. The most common method is carbon black with 20% by weight of platinum put on it (Pt/C), which may generate one of the lowest overpotentials (46 mV at 10 mA cm^–2^) in alkaline circumstances. Pt/C catalysts are frequently utilized as a standard for catalysts created for the HER reaction because of their superior outcomes and performance. Due to its lower cost and similar electronic structures, Ru is always considered a potential candidate to replace Pt for water electrolysis. However, dissolution and stability problems must be thought out.^45,66^

The element titanium is also widely used in electrocatalytic reactions. Titanium dioxide (TiO_2_) is a transition metal oxide that has been extensively studied for its potential application in the hydrogen evolution reaction (HER). However, the traditional crystalline TiO_2_ is generally considered as an inactive material for HER with disappointing performance. This is due to its poor electrical conductivity and unfavorable hydrogen intermediates (H_ads_) adsorption/desorption capability. In order to activate TiO_2_ for HER, theoretical calculations and experimental studies have been carried out to investigate the effect of structural and electronic properties on the hydrogen adsorption free energy (Δ*G*_H*_). The results suggest that the hydrogen adsorption free energy could be optimized through tuning the structural and electronic properties of TiO_2_. For example, amorphous TiO_2_ with disordered atom arrangement can offer a larger amount of catalytic active sites than their crystalline counterparts.^67^

**Figure 5:**
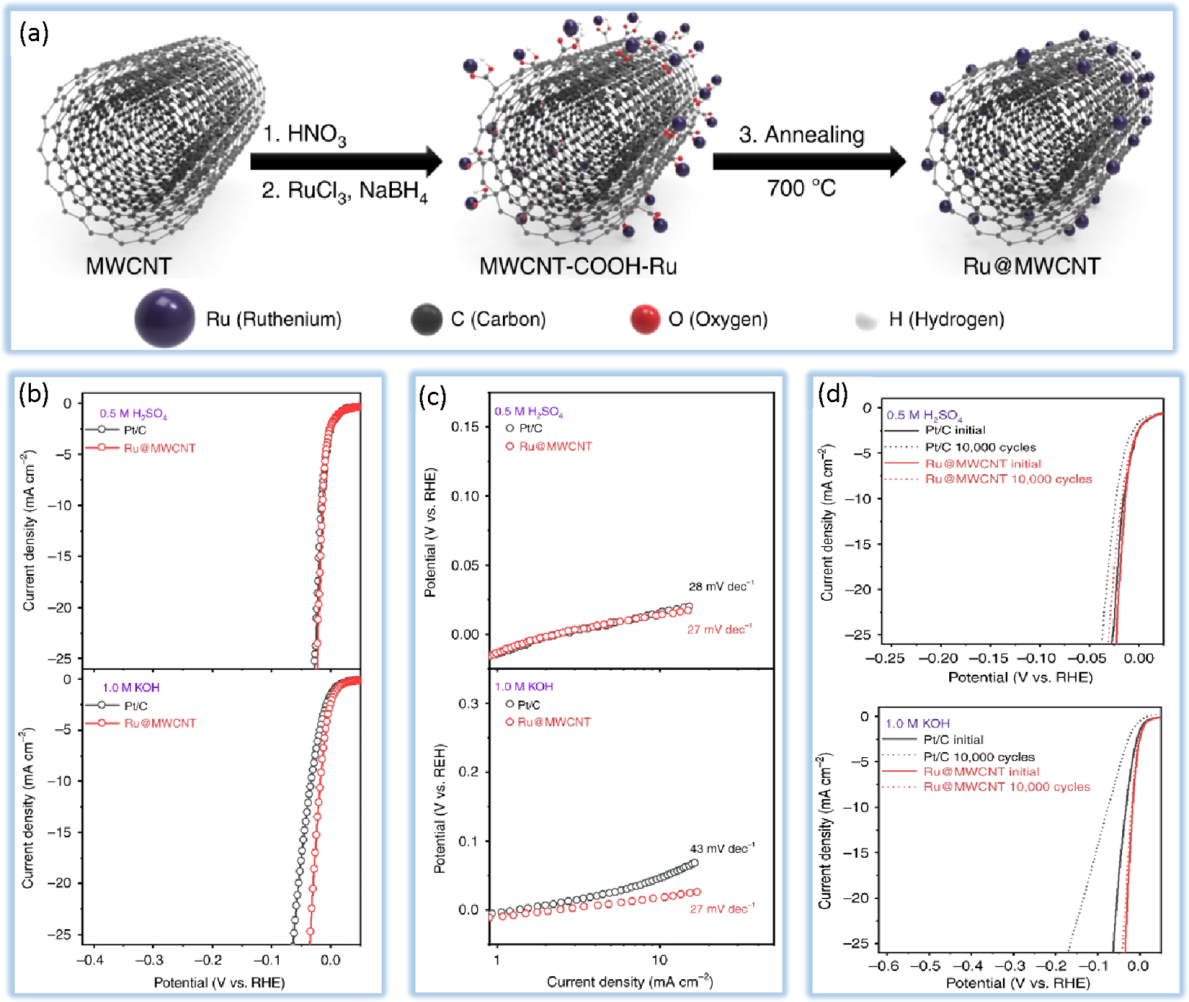
(a) Schematic illustration of the process steps for forming Ru@MWCNT catalyst; (b, c) electrochemical HER performance of the Ru@MWCNT and Pt/C catalysts; polarization curves and corresponding Tafel plots in N_2_-saturated 0.5 M aq. H_2_SO_4_ solution and 1.0 M aq. KOH solution. Scan rate: 5 mV s^–1^; (d) comparison of electrochemical HER; the polarization curves were recorded before and after 10 000 CV potential cycles. Data taken from ref (68).

Kweon et al.^68^ described an inexpensive and simple approach for producing homogeneous ruthenium (Ru) nanoparticles as an effective HER electrocatalyst by depositing Ru on multiwalled carbon nanotubes (MWCNTs) (Figure 5a). According to Figure 5b and Figure 5c, the synthesized electrocatalyst outperforms commercial Pt/C with low overpotentials of 13 and 17 mV at 10 mA cm^–2^ in 0.5 M aq. H_2_SO_4_ and 1.0 M aq. KOH, respectively. Furthermore, the catalyst has exceptional stability in both media, revealing nearly zero during cycling (Figure 5d). In this study, DTF calculations indicate that Ru–C bonding is the most likely effective area for the HER.

In electrocatalysts, surface properties play a decisive role in intrinsic activity and adaptability. Bao et al.^69^ demonstrated a simple approach for thermally doping Ru/C with atomically scattered cobalt atoms. The generated amorphous shell containing Ru–Co sites on the Ru/C electrocatalyst by a surface-engineering approach considerably increased HER activity and stability in this study. Similar to Pt/C but considerably better, the Co_1_Ru@Ru/CNx electrocatalyst displayed an overpotential of 30 mV at 10 mA cm^–2^. This excellent information shows that this obtained electrocatalyst is one of the best HER catalysts for the alkaline environment that has been proposed so far. The other way to reduce the loading of noble metals is to alloy them with inexpensive metals.^70^ The ideal method is to utilize one or more metals that have an extra favorable influence on the material’s catalytic activity. Some Pt-based alloys can be made with Pt–M crystalline nanoparticles (M = Co, Fe, Cu, Ru, Au). In this method, we substitute inexpensive metals for Pt, intending to increase synergy and electrocatalytic performance. There are two key explanations for the improved performance of these materials over commercial Pt nanoparticle-based catalysts. The first explanation is the “ effect of ligand”, which relates to the electronic characteristics of one transition metal’s active sites close to another metal’s active sites. Another reason is the “effect of lattice strain” on the Pt–Pt bond distance because of the mixture of other metals, which causes changes in the Pt metal’s d-bond center.^71^ This factor can be changed by combining Pt with other metals in the network to provide the best catalyst properties. These findings were confirmed by other research groups.^72^ The ability to modify the structure on the surface is essential for enhancing electrocatalytic activity among various noble metal-based electrocatalysts for HER. Doping noble metal-based materials with other metal components has recently been discovered to be another efficient method of increasing the electrocatalytic activity of HER while minimizing the quantity of noble metal utilized. Electrochemical analysis of HER showed that doping, especially the combination of S and N in the noble metal, improves the electrocatalytic behavior and increases the stability. Naveen et al.^73^ reported a Pd nanoparticles (NPs) electrocatalyst supported on a carbon sphere nanoarchitecture doped with sulfur (S) and nitrogen (N) atoms (PdSNC), which is designed by using a palladium–rubeanic acid (Pd–RA) coordination polymer as a precursor, which was then calcined of materials for the HER electrocatalyst. Doping S and N into carbon structures increased the electronic structure and strengthened the affinity of the PdNPs in this study, revealing improved electrochemical performance of the electrocatalyst. By modulating the electronic structure and stabilizing Pd nanoparticles, the placement of dual heteroatoms (S and N) in carbon tissue enhanced stable electrocatalytic activity as an alternative to expensive commercial Pt/C. The modified PdSNC demonstrated a current of 10 mA cm^–2^ at 0.030 V, which is significantly higher than Pd/C and comparable to Pt/C. This strategy used by researchers (doping and Pd/C formation) is a promising approach to designing carbon/noble metal composites with heteroatoms to enhance electrocatalytic performance. In electrocatalysts, surface properties play a decisive role in intrinsic activity and adaptability.^74^ Noble metals provide exceptional benefits for water-splitting processes, but their high price and scarcity remain substantial barriers to broad industrial growth. It should also be mentioned that the usage of noble metals is required for many important applications.^35,75^ As a result, it is possible to infer that the most promising strategy, particularly for large-scale applications, is the creation of electrocatalysts based on cheap and readily available materials. The HER performances of PGM-free catalysts are summarized in Table 2.

**Table 2:** HER Performance Summary of PGM-free Materials Catalysts

material	structure	Tafel slope (mV dec^–1^)	η (mV vs RHE) for *J* = 10 mA cm^–2^	refs
NiCoN	nitride	105.2	145	(76)
Ni_3_N–NiMoN	nitride	64	31	(77)
MoSe_2_/NiSe_2_	selenide	46.9	249 mV at 100 mA	(78)
Ni_0.7_Co_0.3_Se_2_	selenide	31.6	108	(79)
NiMoNx/C	nitride	35	78 mV at 100 mA	(80)
Mo–Fe–Se–CP	selenide	57.7	86.9	(81)
Ni_3_N/VN–NF	nitride	47	56	(82)
Fe_2_P/Fe	phosphide	55	191	(83)
CO_2_P/CP	phosphide	60	174	(83)
CO_2_P	phosphide	101	406	(84)
MO_2_N/NC	nitride	115.7	217	(85)
FeP	phosphide	37	50	(86)
V_8_C_7_@GC NSs/NF	carbide	34.5	38	(87)
WN/CC	nitride	57.1	130	(88)
porous CoP_2_	phosphide	67	56	(89)
MoNx	nitride	114	148	(90)
VMoN	nitride	60	108	(91)
CoNiSe_2_/NF	selenide	40	87	(92)
CoNiSe/NC	selenide	66.5	100	(93)
Mo_2_C/C-900	carbide	52	114	(94)
nanoMoC@GS	carbide	43	124	(95)
MoN	nitride	120	389	(96)
WN–W_2_C	nitride	85	242	(97)
NiCoP holey	phosphide	57	58	(98)
CoP_2_/rGO	phosphide	50	88	(99)

### 4.2 Non-Noble Metal-Based Electrocatalysts

As summarized in the previous chapter, non-noble metal-based electrocatalysts are the only viable option for the future development of large-scale water splitting for energy conversion. According to the study, replacing noble metals with a combination of transition metals and nonmetal elements (S, Se, N, C, and P) has emerged as a potential alternative. Metal sulfides, metal selenides, metal nitrides, metal carbides, and metal phosphides might be mentioned in this group of transition metal-based electrocatalysts with high activity, stability, and affordable pricing.^100^ The following sections summarize the most advanced electrocatalysts selected based on the most common materials and compare each group with Pt-based electrocatalysts.^101^ Mn, Fe, Co, and Ni are some of the most widely used non-noble metal electrocatalysts for HER. As an affordable metal with widespread industrial application and strong HER activity, take Fe as an example (at 10 mA cm^–2^, the overpotential can reach the lowest value of about 260 mV).^102^ Fe is an inexpensive metal that is often employed in industrial settings and has comparatively high HER activity. Pure Fe is employed as a comparison material on occasion, and it can produce diverse findings depending on the preparation process, surface area, morphology, or shape.^103,104^ Unfortunately, there are not many studies done on pure iron as a cathode material. One factor might be the low stability of Fe alone in high-temperature, alkaline conditions. This instability is increased in the no-current mode when the electrolysis is switched off. The low stability and efficiency of Fe cathodes under water-splitting conditions can be reduced by alloying them with one or more metals.^104,105^ These objective characteristics are heavily influenced by the kind of coalloyed metal, overall composition, and preparation technique. Steel materials have historically been utilized as dual-function catalysts in overall water splitting, despite the fact that their catalytic activity, particularly for HER, is significantly inferior to that of the most sophisticated catalysts.^106^ Surface modification appears to be a viable method for dramatically increasing the activity of steel-based materials.^107,108^ In one study, Kim et al.^108^ studied a self-activated nanoporous anodic stainless steel electrode with better catalytic performance for the hydrogen evolution reaction.

Etched and anodized stainless steel (EASS) is created in this technique by employing etched 304 stainless steel foil with an uneven surface and then thermal annealing (650 °C, Ar and H_2_ mixture, 1 h). The synthesis method increases the electrode’s surface area, exposing a large number of active sites and oxygen vacancies. The improved material displayed an overpotential of 370 mV at 10 mA cm^–2^, almost 100 mV less than the original stainless steel (466 mV). In addition, the overpotential is decreased to 244 mV by self-activation after 10 000 LSV cycles due to the creation of a catalytically active metal hydroxide layer on the surface of the electrode. Although significant progress has been made in this field, as far as we know, the most active steel-based electrocatalysts still need to be doped with more active substances, such as Ni, Mo, or P, to achieve an overpotential of only 80–120 mV higher than that observed with commercial Pt/C electrocatalysts.^109^ In addition to stainless steel, the most researched iron-based alloys are the Fe–Co and Fe–Mo groups, which may be utilized as binary alloys or to complement another metal to produce a more complicated system. A high actual surface area Fe–Mo alloy is a potential material for HER.^54−56^

#### 4.2.1 Transition Metal Carbides

**Figure 6:**
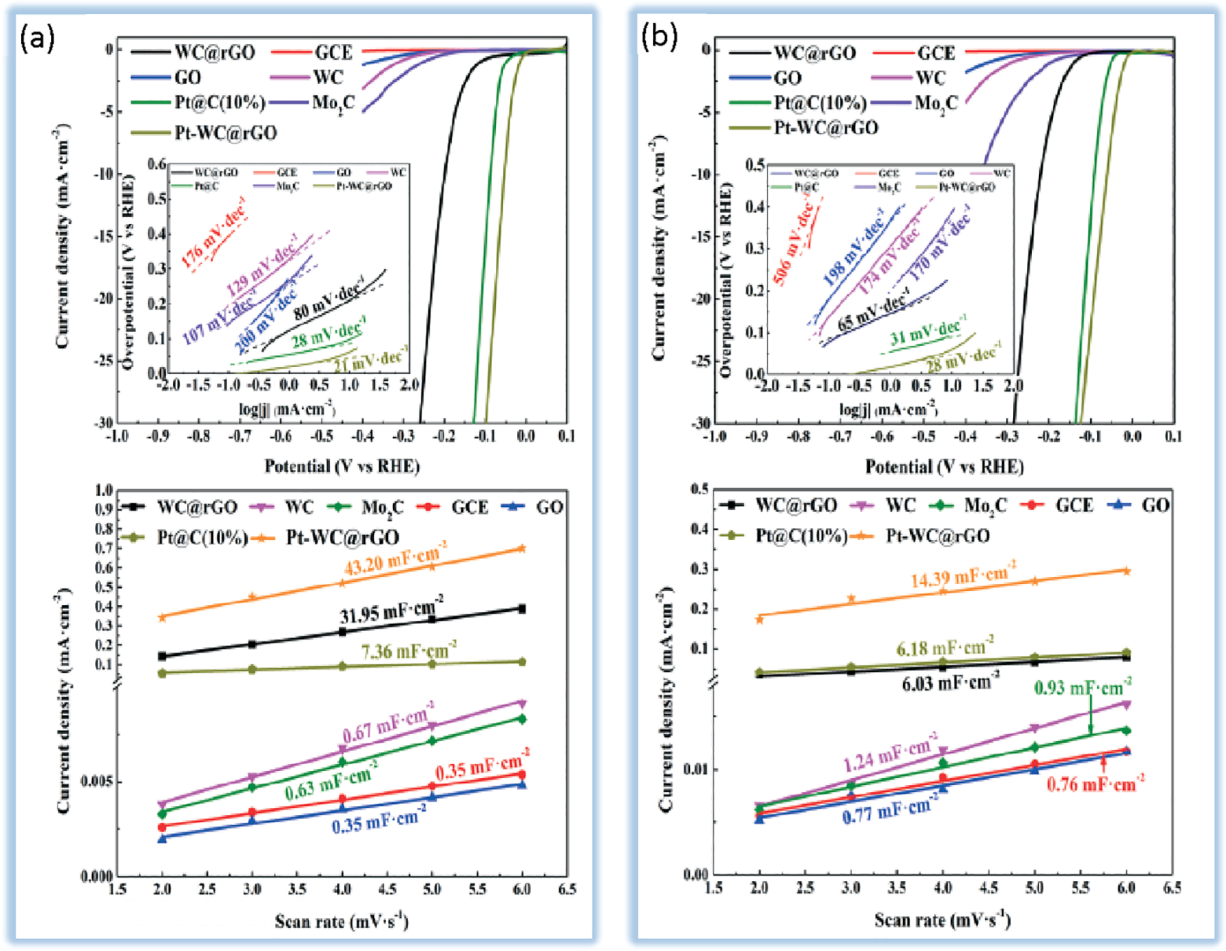
Polarization curves (inset: Tafel plots obtained from the polarization curves), calculated electrochemical double-layer capacitance, and η10 values of corresponding materials in (a) acidic solutions and (b) alkaline solutions. Data taken from ref (113).

Transition metal carbides (TMCs) demonstrate Pt-like properties for HER electrocatalytic activity due to the shift in the d-band center; for this reason, the development of these electrocatalysts as non-noble metal-based electrocatalysts is of great interest.^110^ Group IVb–VIb TMCs are known as transition carbides due to the electronic properties of carbon atoms occupying the metal lattice’s position.^111^ W and Mo are the most common candidates for use in TMCs, which in addition to high electrical conductivity, hydrogen adsorption properties, and d-band electron density state, create the optimal compound that can demonstrate near-Pt electrocatalytic activity for HER electrocatalysis.^112^ WC is one of the first noble metal electrocatalysts for HER electrocatalysis. The W/C ratio substantially influences the electrocatalytic activity of HER in this combination. The electrocatalytic activity of WC, like that of other compounds, is determined by the number of active sites, crystalline phases, and nanoparticle morphology. Lv et al.^113^ adopted a simple approach to create WC@rGO in order to increase HER activity in both acidic and alkaline environments. In order to increase charge transfer and change the size of the WC particle in order to achieve effective and stable HER performance, this study employed the efficiently conductive rGO as a support. The overpotentials of the synthesized electrocatalyst are about 2.5 times smaller than those of the pristine WC to achieve 10 mA cm^–2^ in solutions (acidic and alkaline). As shown in Figure 6a and 6b, the Pt-modified WC electrocatalyst with low Pt accumulation (4% by weight) shows even heightened performance of the electrocatalyst (Tafel slope and η10 of 21 mV dec^–1^ and 54 mV in solution of acid and 28 mV dec^–1^ and 61 mV in solution of alkaline) to commercial Pt/C. The author’s method makes it possible to prepare environmentally friendly, inexpensive, stable, and effective HER electrodes.

It is worth noting that the surface of the electrocatalyst must be free of tungsten oxide or similar compounds to achieve the flawless electrocatalytic activity, as the oxygen species in tungsten carbon disrupt the active sites in the HER electrocatalyst reaction. This is in contrast to transition metal chalcogenides in which the presence of oxygen can improve HER performance. In the interaction of the electrocatalyst with water, the WC surface disrupts and inactivates the formation of a WO_3_ layer.^114^ Although this oxide layer grows at anodic potentials, it should not be present at HER cathodic potentials. Also, its stability in anodic potentials is shallow due to the formation of an anodizing layer. Due to the synergistic effect between WC and Pt, more attention has been focused on using this material as a substrate for Pt electrocatalysts, although its mechanism is still unknown.^115^ Researchers are currently focusing on reducing the Pt content of Pt/WC electrocatalysts with low cost, high efficiency, and good stability for HER.

Mo_2_C also has Pt-like electrocatalytic activity for the HER. In addition, a comparative study showed that molybdenum carbide had better electrocatalytic performance than nitride and boride.^116^ Liu et al.^117^ used CO_2_ as the feedstock to create a self-standing MoC–Mo_2_C catalytic electrode. High performance of the synthesized electrode with low overpotential at 500 mA cm^–2^ was observed in both acidic and alkaline mediums (256 and 292 mV, respectively), due to its hydrophilic porous surface and inherent mechanical strength, the long-lasting lifetime of more than 2400 h, and performance at high temperatures (70 °C). By DFT calculations, the authors show that the superior performance of the HER discontinuous carbide electrode in both acidic and alkaline states is determined by the heterogeneous bonding of MoC(001)–Mo_2_C(101) with Δ*G*_H*_ – 0.13 eV in acid.

The Mott–Schottky phenomenon between a metal with a higher work function and an n-type semiconductor with a greater Fermi level will make electron transmission from the semiconductor to the metal easier. As a consequence, designing optimum H* adsorption active sites using Δ*G*_H*_ is a possibility. Ji et al.^118^ reported that carbonization was used to create MoC nanoparticles embedded in N, P-codoped carbon from polyoxometalates and polypyrrole nanocomposites. Based on an increase in carrier density and an increase in electron transfer rate in MoC after reducing the work function due to the Mott–Schottky effect with n-type domains in N, P-codoped carbon, synthesized electrocatalysts can lead to a current density of 10 mA cm^–2^ at 175 mV with a Tafel slope of 62 mV dec^–1^. In addition, the TOF value at 150 mV is 1.49 s^–1^, as is the long-term stability of H_2_ generation. Metal carbides are a promising choice for HER catalysts in alkaline conditions due to their low cost, high frequency, and the ability of obtaining alkaline activity approaching that of Pt. Regardless of its distinguishing characteristics, the durability of this class of catalysts during intermittent operation remains a critical concern since carbon-based materials are naturally prone to corrosion, even under mild oxidative circumstances. As a result, it is proposed that future study in this field concentrates on strengthening the stability under anodic polarization that can occur after the electrolyzer is turned off.^119^

#### 4.2.2 Transition Metal Phosphides

**Figure 7:**
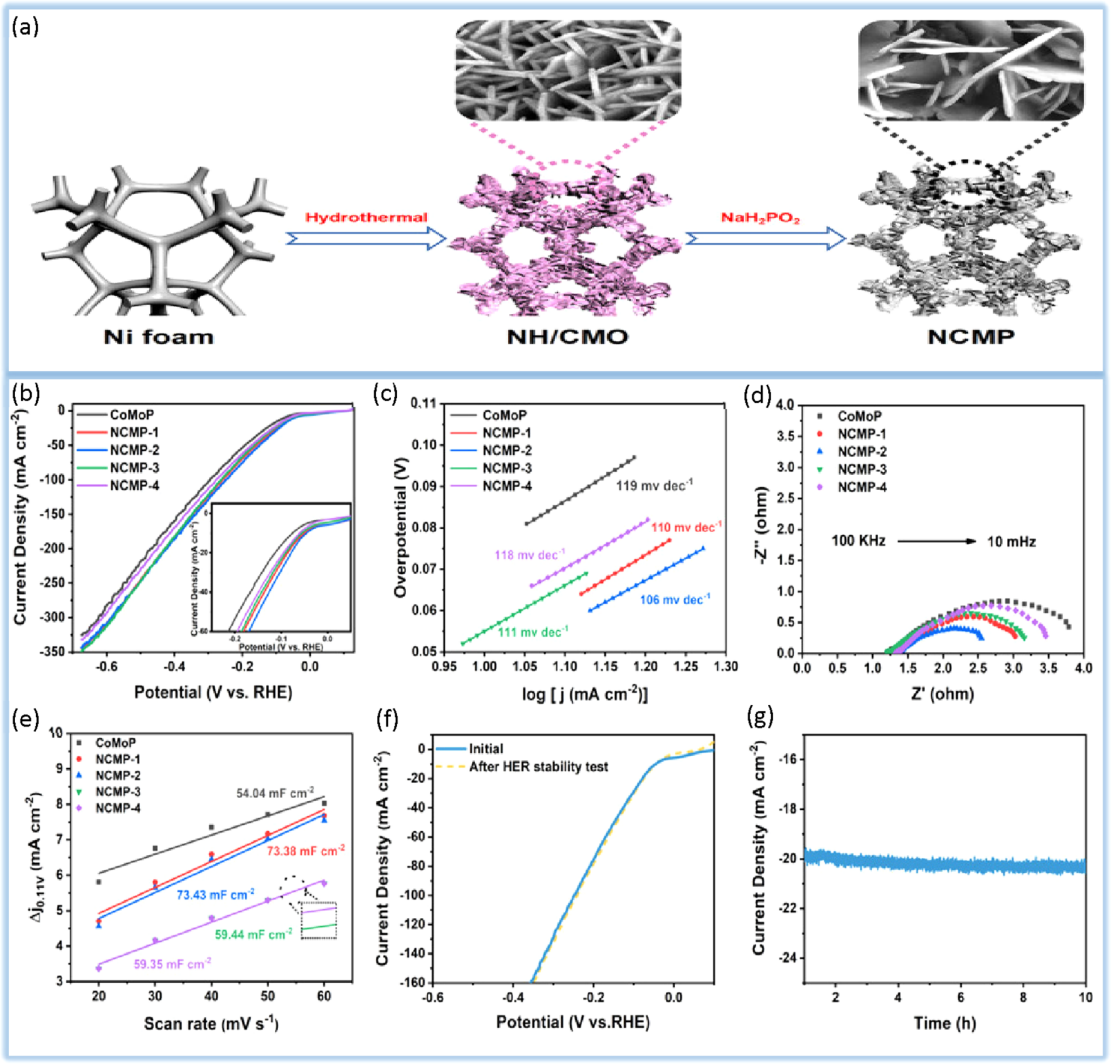
(a) Schematic Illustration of the formation process of NCMP. (b) HER polarization curves of NCMP-*x* (*x* = 1, 2, 3, and 4) and CoMoP in 1 M KOH; inset: partial enlargement of (b). (c) Corresponding Tafel plots. (d) Nyquist plots of NCMP-*x* (*x* = 1, 2, 3, and 4) and CoMoP. (e) Corresponding Cdl of CoMoP and NCMP-*x* (*x* = 1, 2, 3, and 4) at 0.11 V vs reversible hydrogen electrode (RHE). (f) Polarization curves of NCMP-2 before and after 10 h test. (g) Long-term stability of NCMP-2 at a constant overpotential for 10 h in 1 M KOH. Data taken from ref (123).

Transition metal phosphides (TMPs), due to their inherent activity and high stability in both acidic and alkaline environments, have presented themselves as potential candidates for HER electrocatalysis.^120^ In TMPs’ structure, the P atom plays an essential role in electrocatalytic activity due to its excellent conductivity and unique electronic structure. One of the reasons for the superiority of metal phosphides is that the activity of these compounds is not limited to the crystal edge sites. However, HER can also occur in bulk materials. In the TMPs, Ni- and Co-based phosphides are among the lucky members of this group for HER. Computational studies and empirical evidence have always suggested that NiPx alloys are potential candidates for HER electrocatalysis.^121^ Zhang et al.^122^ discovered a multiphase nickel phosphide electrocatalyst composed of porous nickel powder as a matrix with manganese doped into porous nickel powder containing phosphorus powder. After a high-temperature phosphating operation, a multiphase nickel phosphide electrocatalyst (Ni_3_P, Ni_2_P, Ni_12_P_5_) (Mn–NiP) was fabricated with a bimetallic compound. Transition metal phosphides are thought to be affordable and excellent HER catalysts. The activity of the doped catalyst with the right quantity of manganese has risen due to the regulatory influence of doped manganese on the electronic structure of nickel and the electronic contact among nickel and phosphorus. The evolutionary activity of 3Mn–NiP hydrogen in the whole pH range showed outstanding results. The overpotentials required to produce a current density of 10 mA cm^–2^ of 3Mn–NiP were 164 and 77 mV at acidic and alkaline solutions, respectively. Wang et al.^123^ demonstrated a one-step hydrothermal method for synthesizing NiP_2_ with CoMoP nanosheets (NCMP) under low-temperature phosphidation conditions. In the synthesized electrocatalyst, the synergistic effect of two different components, NiP_2_ and CoMoP, is induced. In this study, to find the superior catalyst, the effect of nickel content on the performance of the catalyst is also studied, and it is found that when the nickel dose is 0.02 mM, it shows the most prominent overpotentials of 46 mV at 10 mA cm^–2^. It should be noted that the phosphides of the transition metals are reactive due to the negatively charged P-sites. As a result of this reactivity, a passive layer can quickly form on the electrocatalyst surface, which can completely disrupt the electrocatalytic reaction. Studies have shown that even shallow doses of NiP_2_ can effectively improve the HER performance (Figure 7).

#### 4.2.3 Transition Metal Chalcogenides (Sulfides and Selenides)

Transition metal chalcogenides, relying on properties such as being inexpensive and ease of preparation, are promising candidates to replace noble metals for HER. According to the existing studies, metal selenides show higher catalytic activity than other members of the chalcogenides.^48,124^ The structures of metal sulfides are more straightforward than those of metal electrocatalysts, and their structure must be carefully adjusted for efficient HER operation.^125^ Contrary to popular belief, metal sulfides are not vulnerable to the sulfur position. It is worth noting that these electrocatalysts can perform well in low H coatings, but their electrical conductivity decreases significantly with increasing H coating.^126^

**Figure 8:**
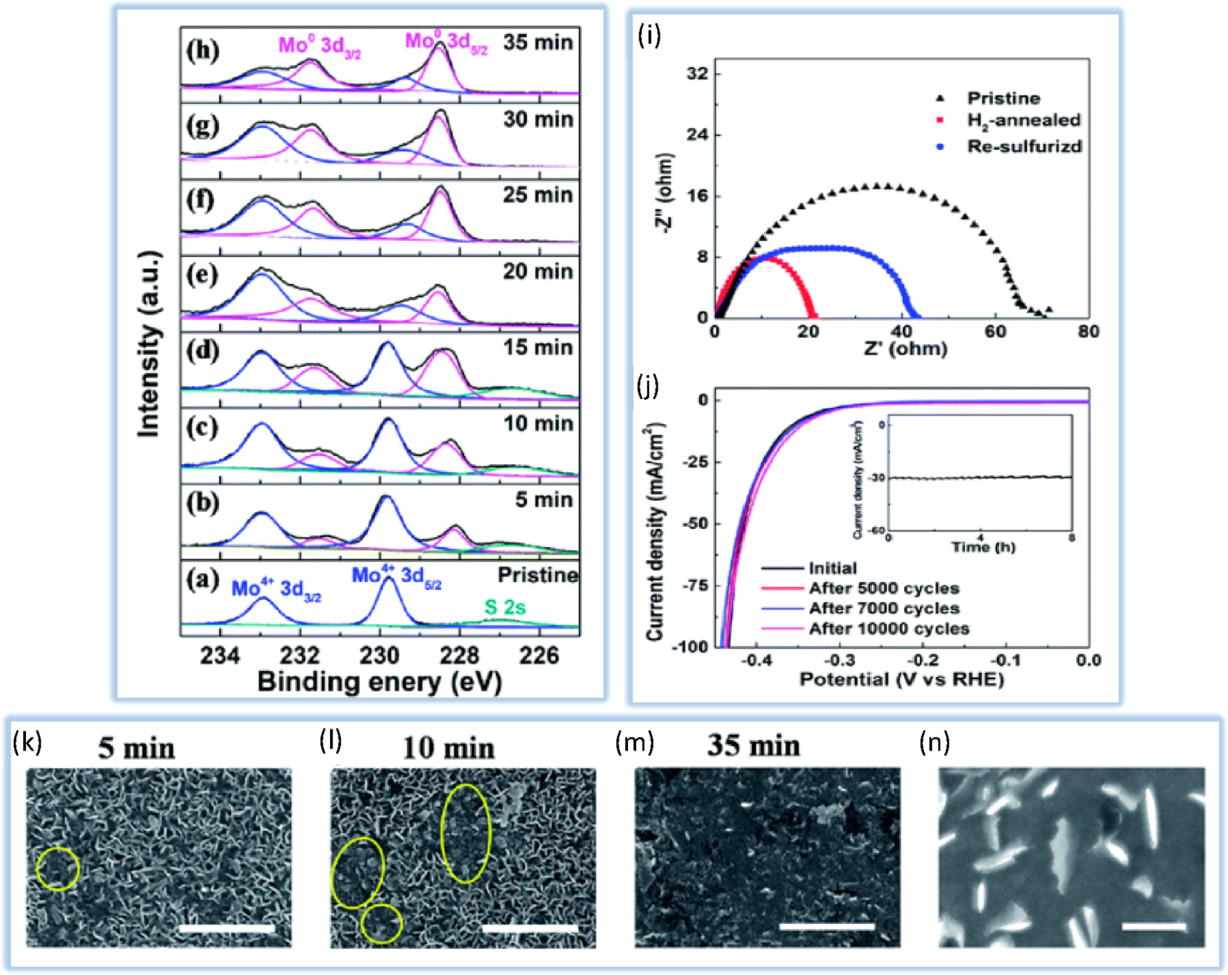
XPS data of the Mo 3d region for (a) pristine and H_2_-annealed vertical MoS_2_ nanosheets for (b) 5, (c) 10, (d) 15, (e) 20, (f) 25, (g) 30 and (h) 35 min, respectively. (i) EIS of pristine, H_2_-annealed, and resulfurized vertical MoS_2_ nanosheets. All of the spectra were collected by scanning from 0.1 to 106 Hz with an overpotential of 0.3 V. (g) Cathodic polarization curves of the H_2_-annealed MoS_2_ for 20 min before and after 5 000, 7 000, and 10 000 cycles, respectively. Inset: time-dependent current density of the H_2_-annealed MoS_2_ for 20 min under a static overpotential of 400 mV for 8 h. (k–n) SEM images of H_2_-annealed vertical MoS_2_ nanosheets for 5, 10, and 35 min, respectively; scale bar: 1 μm. (d) High-magnification SEM image of H_2_-annealed vertical MoS_2_ nanosheets for 20 min, showing rough edges induced by H_2_ annealing; scale bar: 200 nm. Data taken from ref (129).

The shortage of highly efficient and inexpensive catalysts severely hinders the spread of HER on a large scale. Among metal sulfides, molybdenum sulfide is one of the most common candidates for HER electrocatalysts due to its cheapness and flexibility in design.^127^ It can be considered a pioneer in this group of electrocatalysts. In the MoS_2_ electrocatalyst, only the edges and voids S are the active catalytic sites for HER. Therefore, to increase the electrocatalytic activity, it is very important to increase and improve the structure of the edge sites and the S-vacancy.^128^ He et al.^129^ demonstrated HER activity enhancement by combining vertical nanosheets with H_2_ annealing in a modified MoS_2_. Due to a greater number of edges, horizontal MoS_2_ nanosheets with this structural alteration demonstrated stronger HER activity than pure vertical MoS_2_ nanosheets. In justifying the increase in electrocatalytic properties, it should be said that H_2_ annealing further enhanced the HER activity of vertical MoS_2_ nanosheets remarkably. According to Figure 8, XPS results show a minor S:Mo ratio after H_2_ annealing, indicating increased S-vacancy. In the meanwhile, EIS measurements demonstrate that H_2_ annealing speeds up load transfer. SEM images show that H_2_ annealing roughens the edges of MoS_2_ and creates more edge sites, which improves the electrocatalyst behavior for HER.

**Figure 9:**
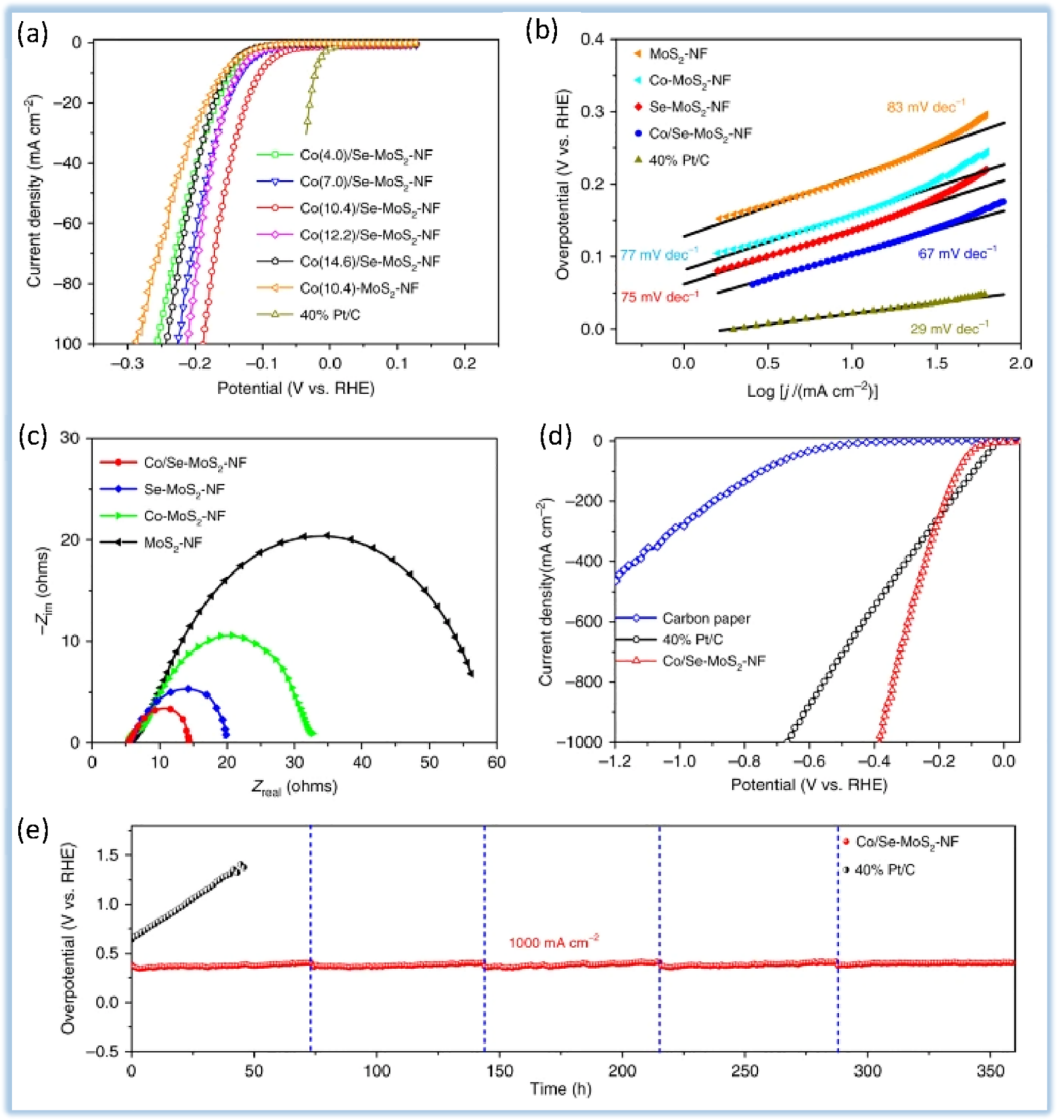
(a) HER polarization curves for Co/Se–MoS_2_–NF with different Co-doping contents in comparison with Co–MoS_2_–NF and 40% Pt/C. All samples are drop-casted on glassy carbon electrode for measurements. The Tafel plots (b) and the electrochemical impedance spectroscopy (EIS) Nyquist plots (c) of Co/Se–MoS_2_–NF sample compared with Co–MoS_2_–NF, Se–MoS_2_–NF, MoS_2_–NF, and commercial 40% Pt/C. (d) HER polarization curves for Co/Se–MoS_2_–NF under large current densities in comparison with 40% Pt/C and carbon paper. Co/Se–MoS_2_–NF and 40% Pt/C are drop-casted on carbon paper for measurements. (e) Chronopotentiometric measurements of long-term stability for Co/Se–MoS_2_–NF and 40% Pt/C at 1000 mA cm^–2^. The vertical dotted lines in (e) mark the time span (every 72 h) of replacing the electrolyte in the 360 h chronoamperometry test. All the HER measurements were conducted in a 0.5 M H_2_SO_4_ electrolyte at 25 °C. Data taken from ref (131).

In an ideal structure, MoS_2_ is a 2D network with six S atoms around each Mo atom in an octagonal configuration. MoS_2_ alone suffers from low catalytic performance for HER.^1,130^ Zheng et al.^131^ proposed a triple-layer MoS_2_ nanofoam structure that encloses the surface selenium and cobalt in the inner layer, whose HER activity is higher than all reported heteroatom-doped MoS_2_. As shown in Figure 9, the Co/Se–MoS_2_–NF-synthesized sample exhibits a much lower overpotential of 382 mV than that of 671 mV over commercial Pt/C catalyst at a high current density of 1000 mA cm^–2^. Also, the high activity can remain stable with the long-term stability of more than 360 h without destruction. According to this study, structure engineering of MoS_2_ via coconfining multielements proposal is a promising and feasible way of developing high-performance and low-cost MoS_2_ electrocatalysts for large-scale HER. DFT calculations show that the Co atoms enclosed in the inner layer of the structure excite the adjacent S atoms. In contrast, the Se atoms enclose the surface of the structure, which allows the creation of comprehensive active sites inside the plate and edge with optimal hydrogen adsorption activity. The strategy in question provides a viable pathway for developing MoS_2_-based catalysts for industrial hydrogen production applications. In this study, Figure 9a shows the HER polarization curves of the Se-doped MoS_2_ nanofoam (Se–MoS_2_–NF) with different Se-doping contents and Co/Se-codoped MoS_2_ nanofoam (Co/Se–MoS_2_–NF) with different Co-doping contents. The onset overpotentials for delivering current densities of −10 and −100 mA cm^–2^ in 0.5 M H_2_SO_4_ electrolyte at 25 °C are plotted for comparison. The Se–MoS_2_–NF sample with 9.1% Se-doping content exhibits the lowest onset overpotentials for both current densities, indicating the highest HER activity. This is because Se doping introduces more edge sites in the MoS_2_ nanoflakes, which are more active for HER. As the Se-doping content increases from 9.1% to 14.6%, the HER activity gradually decreases. This is because excessive Se doping may lead to the formation of overcoordinated S atoms with weakened adsorption of H* at the edge sites. The Co/Se–MoS_2_–NF sample with 10.4% Co-doping content exhibits the lowest onset overpotentials for all three current densities, indicating the highest HER activity. This is because Co doping promotes the formation of more edge sites and also enhances the electronic conductivity of the catalyst. Figure 9b shows the Tafel slopes of the HER polarization curves of the Se–MoS_2_–NF and Co/Se–MoS_2_–NF samples. The Tafel slope is a measure of the rate of electron transfer in the HER reaction. A lower Tafel slope indicates a faster rate of electron transfer, which means a more efficient catalyst. The Co(10.4)/Se–MoS_2_–NF sample exhibits the lowest Tafel slope of 67 mV dec^–1^, which is significantly lower than the Tafel slopes of the Se–MoS_2_–NF (75 mV dec^–1^), Co(10.4)–MoS_2_–NF (77 mV dec-1), and undoped MoS_2_–NF (83 mV dec^–1^) samples. This clearly demonstrates the superior HER kinetics of the Co/Se-codoped MoS_2_ catalyst. Also, Figure 9c shows the results of electrochemical impedance spectroscopy (EIS) measurements of the Se–MoS_2_–NF and Co/Se–MoS_2_–NF samples. EIS measurements are used to measure the charge transfer resistance (*R*_ct_) of a catalyst, which is a measure of the intrinsic resistance to electron transfer. A lower *R*_ct_ value indicates a more efficient catalyst. The Co(10.4)/Se–MoS_2_–NF sample exhibits the lowest *R*_ct_ value of all the samples, which confirms that it is the most efficient HER catalyst.

**Figure 10:**
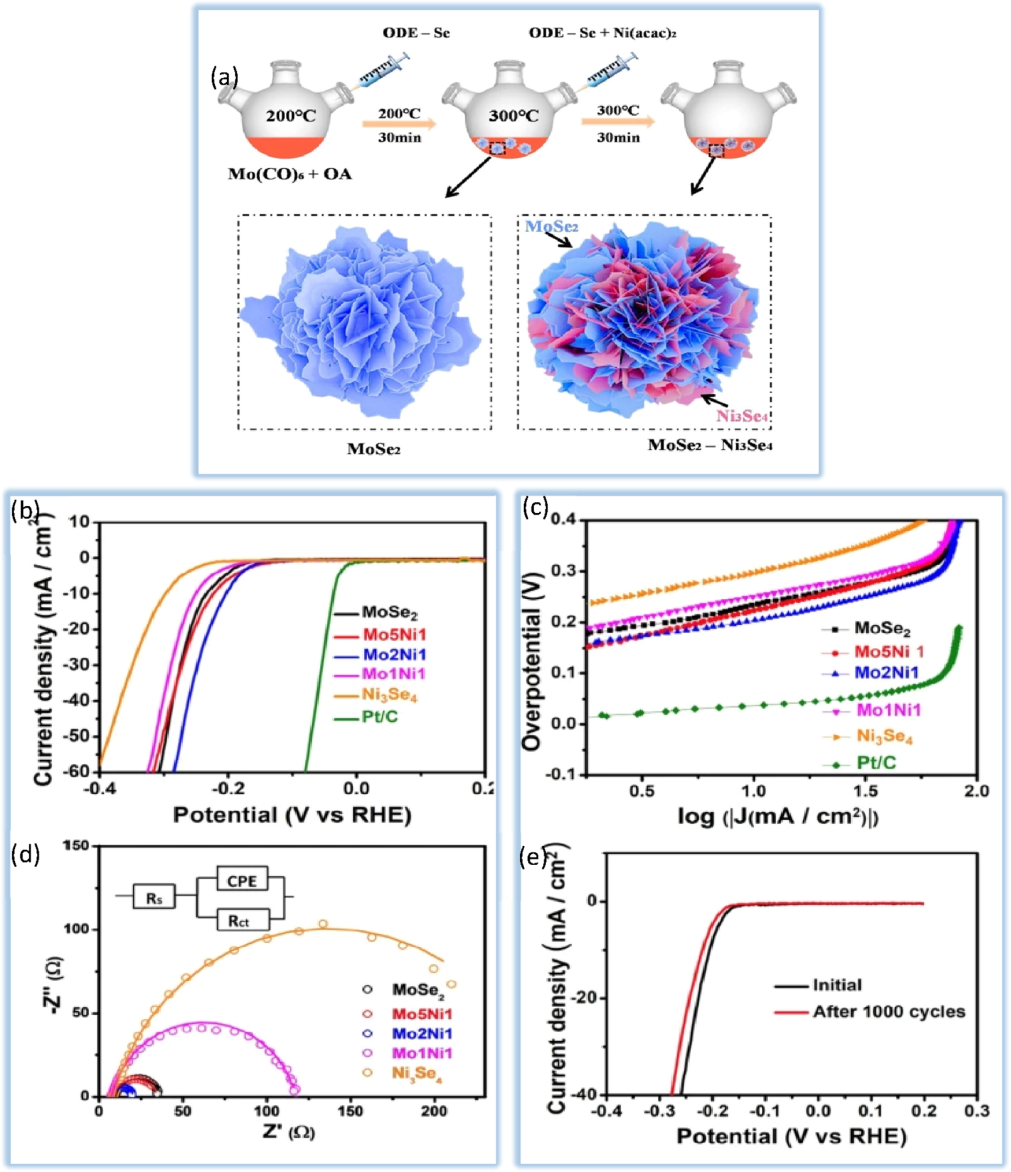
(a) Schematic diagram of the formation of MoSe_2_–Ni_3_Se_4_ hybrid nanoelectrocatalysts. Polarization curves (b) and corresponding Tafel plots (c) of MoSe_2_, Mo_5_Ni_1_, Mo_2_Ni_1_, Mo_1_Ni_1_, Ni_3_Se_4_, and Pt/C. (d) Nyquist plots at an overpotential of 250 mV. (e) Polarization curves of a Mo_2_Ni_1_ sample before and after 1000 cycles. Data taken from ref (139).

Fractal-shaped MoS_2_ has been shown to exhibit enhanced hydrogen evolution reaction (HER) performance compared to its planar counterpart. This is attributed to the increased number of edge sites, which are the active sites for HER. The fractal morphology of MoS_2_ can be induced by nonequilibrium growth conditions, such as rapid heating rates and high Mo precursor concentrations. The enhanced HER activity of fractal MoS_2_ can be attributed to the increased number of edge sites. Edge sites are more reactive than basal plane sites due to their higher exposure to the electrolyte. The higher reactivity of edge sites is due to the weaker bonding between edge atoms and the MoS_2_ lattice. This weaker bonding allows edge atoms to more easily dissociate hydrogen from water molecules. The fractal morphology can be induced by nonequilibrium growth conditions, and the enhanced activity can be attributed to the increased number of edge sites and the higher surface area.^132^ Like metal sulfides, the metal selenides have attracted much attention in the past decade. Because the intended reaction only occurs on the crystal’s edges, selenides’ electrocatalyst activity is greatly determined by the amount of active sites on its surface.^133^ Therefore, catalyst surface optimization seems to be critical for creating active and enriched edge locations. In general, metal selenides, like metal sulfides, follow a similar route due to their propensity to function as an active catalyst for HER in an alkaline environment.^134^ Aside from surface modification, combining a metal selenide with another group(s) (such as phosphides or sulfides) can have a significant impact on the electrocatalyst’s efficiency.^135^ The strategy of this method is based on increasing the number of active sites in the electrocatalyst, which leads to increased electrode activity. MoSe_2_ is considered a promising electrocatalyst for the HER to produce green energy due to its availability and low preparation cost.^136^ Applicable structural changes in the building of this MoSe_2_ can significantly improve its efficiency.^137^ In the past decade, the use of 2D structures and hybrid nanoelectrocatalysts for HER has emerged as efficient and cost-effective electrocatalysts. A MoSe_2_ hybrid with other transition metal dichalcogenides to form an optimal nanostructure is an effective way to increase HER electrocatalytic activity to replace a Pt electrocatalyst.^138^ Wu et al.^139^ adopted a seed-induced solution technique to create MoSe_2_–Ni_3_Se_4_ hybrid nanoelectrocatalysts with a flowerlike shape (Figure 10. In the structure, the Ni_3_Se_4_ component instinctively tends to nucleate instead of independent nucleation to form individual nanocrystals, growing on the surfaces of very thin MoSe_2_ nanoparticles to form an efficient hybrid nanostructure. The HER catalytic activities of MoSe_2_–Ni_3_Se_4_ hybrid nanoelectrocatalysts with varied Mo:Ni ratios are compared. The comparison of MoSe_2_–Ni_3_Se_4_ hybrid nanoelectrocatalysts with varied Mo:Ni ratios reveals that Mo:Ni ratios impact HER activities. The MoSe_2_–Ni_3_Se_4_ hybrid has a Mo:Ni molar ratio of 2:1 and increased HER characteristics, with a Tafel slope of 57 mV dec^–1^ and an overpotential of 203 mV at 10 mA cm^–2^. It should be noted that only the triangular prism phase edge locations have HER electrocatalytic properties and the base plate, which usually does not have defective/unsaturated locations, is inactive. In addition to the alkaline medium, metal selenides have been successfully tested in acidic media.

#### 4.2.4 Transition Metal Nitrides

**Figure 11:**
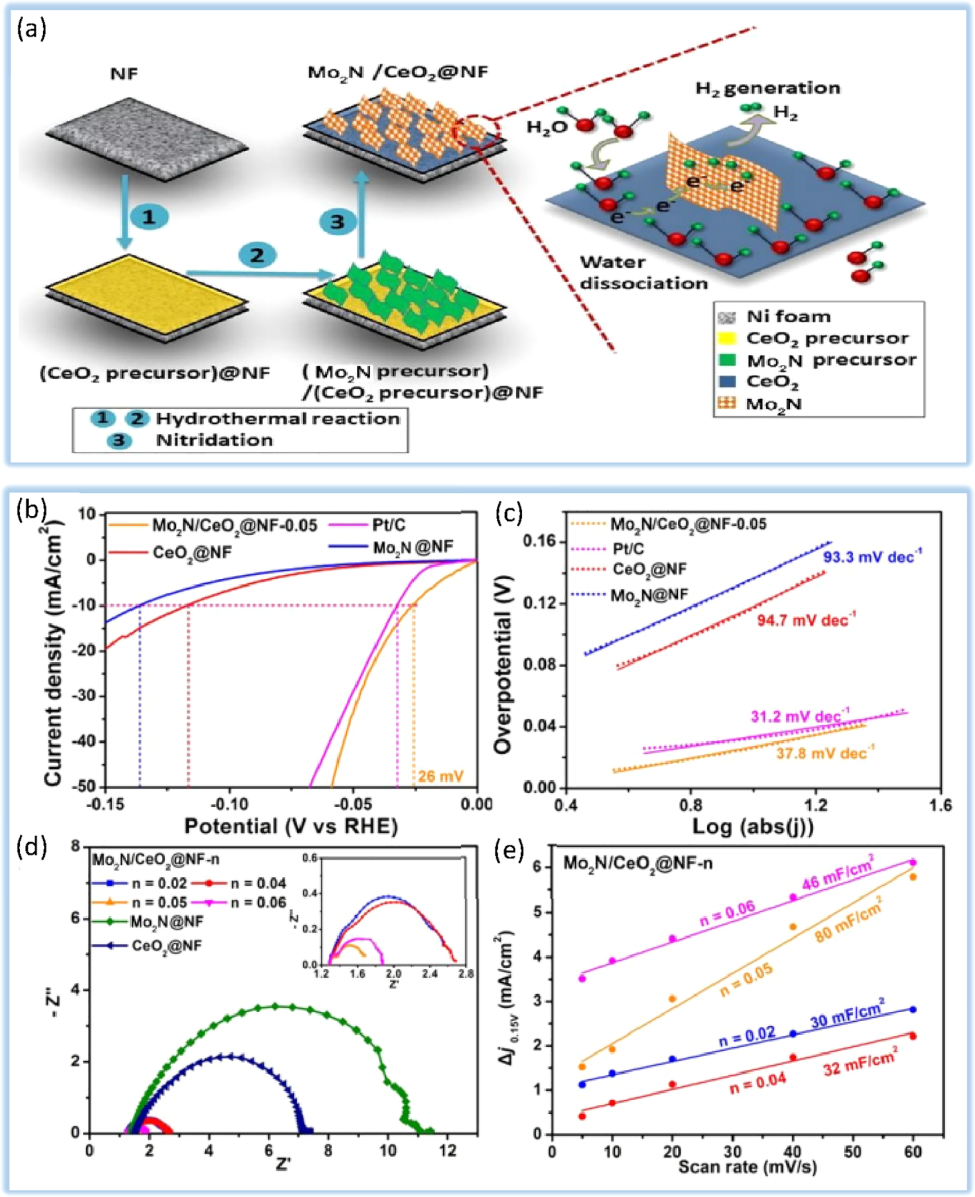
(a) Schematic illustration of the fabrication process of Mo_2_N/CeO_2_@nickel foam and the catalytic process. (b) Current vs overpotential plots for Mo_2_N/CeO_2_@NF-0.05, CeO_2_@NF, Mo_2_N@NF, and Pt/C. (c) Tafel plots for Mo_2_N/CeO_2_@NF-0.05, CeO_2_@NF, Mo_2_N@NF, and Pt/C. (d) Nyquist plots for Mo_2_N/CeO_2_@NF-*n* (*n* = 0.02, 0.04, 0.05, and 0.06), CeO_2_@NF, and Mo_2_N@NF. Inset, enlarged view of Nyquist plots for Mo_2_N/CeO_2_@NF-*n* (*n* = 0.02, 0.04, 0.05, and 0.06). (e) Current density as a function of scan rate of various electrodes, where the slope represents Cdl. Data taken from ref (144).

Transition metal nitrides (TMNs), known as transition alloys, have recently been proposed as efficient HER electrocatalysts as alternatives to noble metal electrocatalysts due to their exceptional electrical conductivity, corrosion resistance, mechanical robustness, and high electrochemical stability.^140^ Metal nitrides are particularly appealing for future investigation due to their exceptional properties. Direct nitridation is the traditional way of producing metal nitrides. Heating an NF (nickel foam) in an NH_3_ atm, for example, can provide an active Ni_3_N/Ni foam electrocatalyst with exceptional qualities (overpotential 121 mV at 10 mA cm^–2^ and even stable in an alkaline environment, 1 M KOH, 32 h).^141^ TMNs containing Mo or Ni can achieve Pt-like activity in alkaline environments.^142,143^ Wang et al.^144^ published a self-supporting electrocatalyst preparing the growth of a MO_2_N layer on a CeO_2_ layer deposited on NF. According to Figure 11a, the synthesized MO_2_N/CeO_2_@NF showed an attractive catalytic activity for the HER at 1.0 M KOH even more efficiently than the Pt/C electrode and relatively low overpotential of 26 mV for a current density of 10 mA cm^–2^. The stability of this electrocatalyst for a long time (24 h) was also evaluated. The results offer great hope for building an inexpensive and stable TMNs electrocatalyst for a water-splitting reaction.

This study discusses the current density versus overpotential plots (Figure 11a) obtained from linear sweep voltammetry (LSV) measurements. It compares the HER activities of different electrodes, including Pt/C, CeO_2_@NF, Mo_2_N@NF, and Mo_2_N/CeO_2_@NF-*n*. The text highlights the overpotentials and current densities at 10 mA/cm^2^ for each electrode, demonstrating the performance of Mo_2_N/CeO_2_@NF-0.05 as the electrode with the smallest overpotential for HER in alkaline solution, comparable to Pt. Authors provides Tafel plots (Figure 11b) to analyze the kinetics of the HER for the different electrodes. The Tafel slopes obtained from these plots are compared, with Mo_2_N/CeO_2_@NF-0.05 and Pt/C showing smaller slopes than CeO_2_@NF and Mo_2_N@NF. This comparison suggests that Mo_2_N/CeO_2_@NF-0.05 is as efficient as the Pt electrode for HER in alkaline solution. Additionally, this report includes Nyquist plots (Figure 11c) obtained from electrochemical impedance spectroscopy (EIS) measurements. The analysis of the Nyquist plots indicates that Mo_2_N/CeO_2_@NF-0.05 exhibits the fastest electron transfer in the HER process, as reflected by the smallest semicircle radius. This observation aligns with the electrode’s lowest Tafel slope and suggests that Mo_2_N/CeO_2_@NF-0.05 has the best HER activity among the different Mo_2_N/CeO_2_@NF-*n* electrodes.

The fabrication of Ni-based electrode materials has attracted much attention due to their suitable activity and good stability to replace expensive Pt for producing molecular hydrogen through the electrolysis of water. Supporting elements such as carbon are commonly utilized in this context to boost electrical conductivity and catalytic activity. Balaji et al.^145^ observed a particular pathway for fabricating carbon-backed Ni_3_N/Ni as an efficient HER electrocatalyst in both 0.5 M H_2_SO_4_ and 1 M KOH environments. By providing intrinsically active sites, carbon support can effectively enhance the electronic structure of Ni_3_N/Ni. Superior electrical conductivity and charge transfer rate are demonstrated by the optimized Ni_3_N/Ni@C electrocatalyst. The TMNs composite also showed improved electrocatalytic behavior with a low overpotential of 163 and 172 mV at 10 mA cm^–2^ and durability over 1000 cycles in acidic and alkaline solutions for HER application. In TMNs, the density of states in the d-band of the corresponding metal is regulated by the nitrogen atom.^143,146^ Therefore, there is a minor deficiency in the occupation of the d-band. In this respect, they can compete with TMCs. An exciting feature of TMNs is that they can be used in alkaline environments with high stability.

Kamran et al. reported the development of a novel nanoheterostructured catalyst for the HER in alkaline electrolytes. The catalyst, Ni(OH)_2_@Ni–N/Ni–C/NF, is composed of Ni(OH)_2_ nanoparticles embedded in an outer layer of Ni–N/Ni–C. The nanoheterointerfacing between Ni(OH)_2_ and Ni–N/Ni–C phases is shown to enhance the HER activity significantly compared to monophasic Ni(OH)_2_. The authors attribute the enhanced HER activity to the interphasic synergy between Ni(OH)_2_ and Ni–N/Ni–C phases. Ni(OH)_2_ is a strong water-splitting promoter, but it has a strong affinity for hydrogen, which hinders the hydrogen evolution process. Ni–N/Ni–C phases, on the other hand, have labile electronic properties and can facilitate the dissociation of hydrogen from adsorbed hydrogen species. The nanoheterointerfacing between Ni(OH)_2_ and Ni–N/Ni–C phases allows for the efficient transfer of electrons from Ni(OH)_2_ to hydrogen species on Ni–N/Ni–C phases, leading to enhanced HER activity. The authors demonstrated the superior performance of Ni(OH)_2_@Ni–N/Ni–C/NF by comparing its HER activity to that of monophasic Ni(OH)_2_ and other benchmark catalysts. Ni(OH)_2_@Ni–N/Ni–C/NF exhibited the lowest overpotentials for delivering current densities of −10 and −100 mA cm^–2^ in 1 M KOH compared to monophasic Ni(OH)_2_ and other catalysts. The Tafel slope of Ni(OH)2@Ni–N/Ni–C/NF was also lower than that of monophasic Ni(OH)_2_, indicating a faster reaction rate. This work further demonstrated the stability of Ni(OH)_2_@Ni–N/Ni–C/NF in alkaline electrolytes by subjecting it to cyclic voltammetry (CV) and chronopotentiometry (CP). Ni(OH)_2_@Ni–N/Ni–C/NF exhibited good stability during CV and CP, with minimal degradation over time.^147^

### 4.3 MOF-Based Electrocatalysts

The use of metal–organic frameworks (MOFs) in electrocatalytic, photocatalytic, and chemocatalytic processes makes them a strong choice for extremely effective HER.^148^ Research on MOF-based HER catalysts involves a wide range of porous materials including as zeolite, polymer, carbon, and composite materials.^149^ These species have evolved porosity with high specific surface area, flexible crystalline porous frameworks, and big pore volume, which is the primary property of their applicability in the HER process. Aside from the intrinsic features indicated, external changes to the main MOFs, such as the loading of active nanoparticles and the bonding of functional surface species, are useful in increasing the activity of the catalysts.^150^ However, MOF-based catalysts are more attractive and diverse than other types of porous catalysts due to the various compounds of metal centers and organic ligands. These compounds, like most other porous catalysts, can be utilized in “pristine”, “precursor”, or “derived” forms for the dispersion or separation of active catalytic species added to porous networks. According to research studies, the combination of nanoparticles and MOF compounds can produce amazing synergistic effects that lead to much better performance for the HER.^151^

As mentioned, electrocatalytic HER is usually achieved by establishing an electrical current due to a potential difference through an aqueous electrolyte to produce O_2_ at the anode through the OER process and produce H_2_ at the cathode side through the HER process. The half-reaction at the cathode side is the focus of most electrocatalytic HER research. However, in the case of water splitting, the performance of MOF-based catalysts at HER and OER must be compared.^152^ MOFs are frequently employed as supports or precursors; MOF-based derivatives or composite materials may retain the original MOF’s porous structure, but the pore volume and specific surface area are decreased. Reduced porosity in MOF derivatives is caused by the collapse of the crystalline porous structure caused by breaking coordination bonds and eliminating noncarbon components at high temperatures.^153^ When MOF is utilized as a support, it usually results in the colonization of pores by guest particles.^154^ The reduction in porosity in this example is proportional to the size and dispersion of the guest particles.^155^

**Figure 12:**
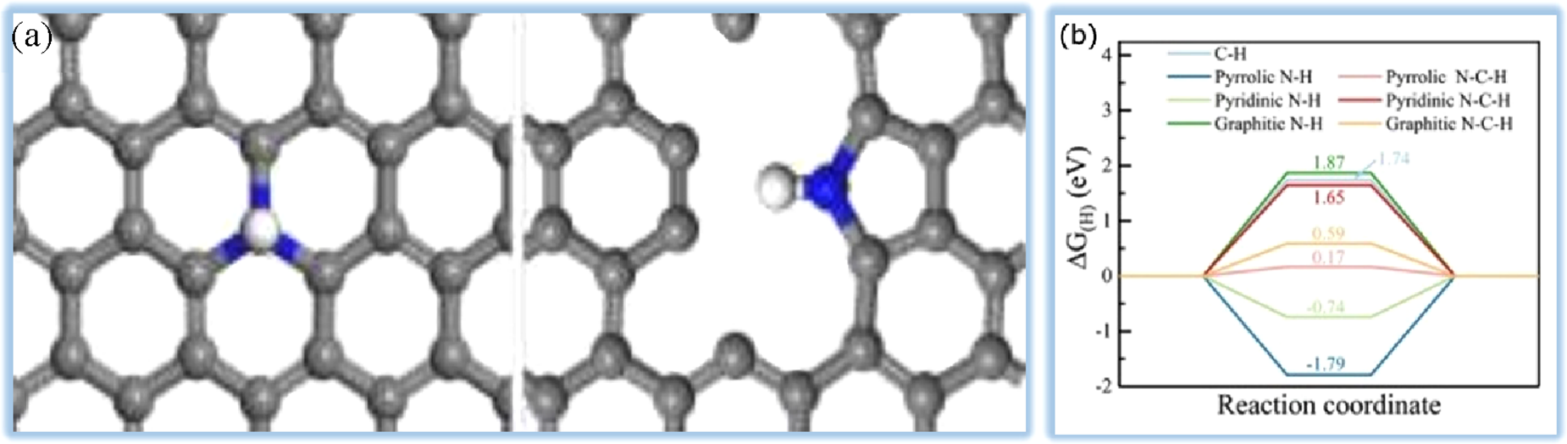
(a) Heteroatom-doping process on graphitic carbon. (b) Nitrogen-doped vertical graphene as a metal-free catalyst for HER exhibits a good activity and an excellent stability, which can be ascribed to the optimization of Δ*G*_(H)_ by N doping and the robust structure of VG. Data taken from ref (159).

However, the performance of MOF derivatives is not always compromised by lower porosity. It is important to note that MOF heat treatment is frequently used to produce MOF derivatives with particular chemical makeups and structures, such as metal oxides, metal carbides, metal nitrides, and metal sulfides. Yan et al.^156^ synthesized nickel–cobalt bimetallic phosphide nanotubes from MOF-74–Co_*x*_Ni_*y*_ bimetallic for electrocatalytic water distribution. The specific surface area of pristine MOF reduced from 1100 to 55.6 m^2^ g^–1^ after oxidation and phosphorylation. Encapsulation of guest molecules, on the other hand, tries to add more catalytically active sites into the original MOF or to increase the synergy between guest molecules and host materials. In 2017, Hao et al.^157^ published the introduction of MoS_2_ quantum dots and graphene into UiO-66–NH_2_ (Zr-containing MOF) for HER. The specific surface area of the original MOF is 712.3 m^2^ g^–1^, which decreases to about 457.24 m^2^ g^–1^ after loading MoS_2_ (5.0% by weight) and graphene. Zhen et al.^158^ improved the Ni nanoparticle-loaded MOF-5. After synthesis, due to the much-dispersed nanoparticles, the corresponding specific surface area reduced slightly (from 2973 to 2961 m^2^ g^–1^). These MOF-based materials mentioned above perform better in HER catalysis than their primary precursors (Figure 12).

## 5 Summary and Perspectives

Hydrogen is a favored alternative to fossil fuels since it has the highest gravitational energy density and produces no pollution, making it a clean and sustainable energy source. In the meanwhile, establishing water-dividing cells as an energy-efficient conversion mechanism is critical to hydrogen generation. However, due to the sluggish reaction kinetics of the OER and HER for the high overpotential, the energy efficiency of water electrolysis is low, and its practical use is hampered. HER is most likely the simplest and most direct method of producing high-quality and selective hydrogen. Pt has long been a suitable HER electrocatalyst, but its limited supplies and high cost make developing HER impracticable. Furthermore, since Pt must be utilized in combination with a carbon catalyst support, the mechanical stability of the electrocatalyst is a feature that might raise operating expenses. Pt recycling is another issue that plagues Pt-based systems. As a result, a shift in the approach of classic electrocatalysts appears to be unavoidable. However, enhancing present Pt electrocatalysts as a solution can drastically lower operating expenses. Practical methods for HER electrocatalysts have recently been established using non-noble metals and related compounds. To assist the practical use of water distribution in enterprises, the design of effective catalysts is critical in OER and HER to limit possible overcapacity, boost stability, and improve efficiency. The most common electrocatalytic activity parameters, such as overpotential at constant current density (10 mA cm^–2^) and Tafel slope, along with at least one of the classical stability tests, were selected as the main comparison parameters. The main parameters affecting the resulting catalytic activity can be summarized as follows:

(a) The incorporation of transition metals into the electrocatalyst design can be advantageous. Although these beneficial effects are tunable, optimal synergistic effects between various metals boost electrocatalytic capabilities.
(b) The catalyst core/shell design is a realistic and practical strategy to enhance the active sites for HER electrocatalysis while making the material feasible for development. It is particularly effective and successful in increasing active sites around the borders of two-dimensional layered structures. However, although the core/shell design enhances catalytic performance, the overall structure may be inadequate for industrial operation.
(c) A high-level carbon catalyst is required for support. To obtain adequate performance, even with Pt, a sufficient carbon catalyst support must be utilized (and not just low overpotential). However, the physical and chemical structure of carbon is undeniably important in electrocatalytic efficacy.
(d) Transition-based chemicals such as sulfides, selenides, phosphides, and carbides are the most promising options.
(e) The employment of transition metals in electrocatalytic architecture, similar to doping, can be advantageous.
(g) The substrate surface plays an important role, especially for electroactive monolayers or ultrathin films. This effect is seen on both the smooth surface and the particle coating.
(H) Planar deformation and strain engineering can assist activate putative electrocatalytic sites on base surfaces.
(i) MOF-based materials demonstrate significant activity in the HER due to their high porosity, controlled porosity, and suitable structure. These materials are utilized as electrocatalysts for HER for several reasons. First, they offer an opportunity to enhance and replace expensive noble metal-based catalysts with more affordable metals. Second, they aid in reducing the necessary overpotential for HER, thereby improving the overall efficiency. Lastly, MOF-based materials contribute to improved reaction kinetics, enabling more efficient HER processes. Overall, the unique characteristics of MOFs make them promising candidates for HER electrocatalysis.

Despite significant advances in understanding electrocatalytic processes and designing suitable electrodes for the HER, there are several challenges to the cost-effective production of large-scale hydrogen by split water electrolysis. To ensure the successful progress of the research, it is necessary to integrate the test protocol in order to be able to compare different materials. In addition, when designing novel cathodic catalysts in the research phase, the ease of preparation and potential scalability for industrial applications should be considered. However, recent interest in a different approach to energy sources, the importance of environmental protection in the future, and economic issues are expected to lead to new advances in the design of active, sustainable, and low-cost HER electrocatalysts for mass commercialization of water-based hydrogen production.
